# Effects of beer addition on the fermentation quality and flavor development of pickled peppers

**DOI:** 10.1016/j.fochx.2025.102845

**Published:** 2025-08-05

**Authors:** Linzhu Li, Wei Su, Yingchun Mu, Yu Jiao, Ling Chen, Huayan Luo

**Affiliations:** a School of Liquor and Food Engineering, Guizhou University, Guiyang 550025, China; b Guizhou Key Laboratory of New Quality Processing and Storage of Ecological Specialty Food, China

**Keywords:** Beer, Pickled peppers, Fermentation quality, Flavor development, Microbial community

## Abstract

This study explored the effects of beer as a fermentation medium additive, with beer addition levels of 25 % and 50 %, on the fermentation quality and flavor development of pickled peppers. Beer addition resulted in higher pH, reducing sugar, and nitrite levels, as well as improved textural crispness, while reducing total acidity and salinity. Microbial community analysis indicated that Firmicutes, Proteobacteria, and Ascomycota were the dominant phyla. Volatile compounds were predominantly categorized into esters, alcohols, aldehydes, and terpenes; collectively, 37 differential volatile compounds were identified, including 24 esters, 5 aldehydes, 3 alcohols, 3 terpenes, 1 acid, and 1 ketone. Moreover, Spearman correlation analysis revealed that *Lactiplantibacillus* and *Wickerhamomyces* exhibited significant correlations with key flavor substances. This study can provide a reference for optimizing the fermentation process of pickled peppers and improving the flavor quality of products.

## Highlights

• Flavor and microbial composition were studied during the fermentation of pickled peppers with added beer
• The addition of beer increased the contents of esters, alcohols, and aldehydes.
• *Lactiplantibacillus* and *Wickerhamomyces* were related to key volatile compounds.
• The addition of beer improved instrumental textural crispness and sensory quality.

## 1 Introduction

Pickled peppers are a unique traditional fermented chili product in Southwest China, cherished by consumers for their distinctive flavor and tender texture. The traditional production of pickled peppers primarily uses fresh red chilies as raw materials: after being cleaned and air-dried, salt, water, white wine, and a small amount of vinegar are added, followed by placement in a pickle jar for natural fermentation at room temperature for 30 days (Tang et al., 2024). During this process, various microorganisms attached to the surface of the chilies work together to endow pickled peppers with their unique flavor (Xu et al., 2020). However, traditional fermentation relies on the indigenous microbial community, which is highly unstable, easily influenced by the environment, and susceptible to contamination by harmful microorganisms. This leads to numerous issues in actual production, including excessive proliferation of harmful microorganisms, insufficient process standardization, texture softening, and odor generation (Liu et al., 2023). In recent years, to realize controllable production and standardized quality of fermented chilies, most studies have centered on the intrinsic system of fermented chilies, such as raw material characteristics, salt reduction techniques, associations between microbial communities and metabolites, and aroma component profiling (Tang et al., 2024; Ye et al., 2022; Zhang, He, et al., 2023), while studies on the addition of nutritional substrates are relatively limited.

The addition of alcoholic substances as medium additives in fermentation systems has been proven to effectively improve the quality and safety of fermented vegetables. For example, Huang, Li, Wu, Tong, et al., 2025 used fermented radishes as the main raw material to explore the effects of different types of Baijiu (Chinese liquor) on the quality of Sichuan pickles. They found that the addition of Baijiu significantly promoted the production of esters, alcohols, and terpenes in pickles, and improved their safety, flavor, and overall quality. Xian et al., 2022 further found that the addition of Baijiu could inhibit membrane-forming microorganisms in Sichuan pickles, thereby enhancing product quality and safety. These studies have confirmed that alcoholic additives can effectively improve the flavor, quality, and safety of fermented vegetables by regulating the microbial community structure and the metabolism of flavor substances, showing good application potential in the field of fermented vegetables. However, most existing studies have focused on high-alcohol-content Baijiu, whose function relies on the bacteriostatic property of high-concentration ethanol and whose composition is relatively simple. In contrast, beer, as a low-alcohol composite substrate, not only contains low-concentration alcohol but is also rich in various nutrients. Yet, its application as a fermentation medium additive in chili fermentation has rarely been reported.

Beer is brewed through yeast fermentation using malt, hops, and water as primary raw materials, and it is rich in nutrients such as carbohydrates, proteins, fats, vitamins, and phenols (Olas & Brys, 2020). Currently, beer is predominantly applied in the field of culinary processing (e.g., in the preparation of beer-braised fish and beer-braised duck) (Liu et al., 2024; Zhang, Xiao, et al., 2023), yet its mechanism of action in fermented food systems remains unclear. In traditional folk practices or small-scale workshop processes, beer is often used to marinate pickled peppers, resulting in a crisper texture and richer flavor. However, such traditional processes lack standardized control, leading to poor stability in product quality, and the mechanisms underlying flavor formation and microbial activity have not been scientifically elucidated. Additionally, most existing studies on fermented peppers employ relative quantitative methods to analyze microbial community abundance, which fail to accurately reflect their actual dynamics. In contrast, microbial absolute quantification technology enables precise quantification of the absolute abundance of communities, providing a more reliable basis for analyzing community composition (Vandeputte et al., 2017).

Based on this, the present study employs beer as a fermentation medium additive to investigate the effects of different beer addition amounts (25 % and 50 %) on the fermentation quality and flavor of pickled peppers. The main objectives are as follows: (a) determination of the optimal beer addition level; (b) exploration of how beer addition influences the flavor and sensory quality of pickled peppers; (c) analysis of the effects of beer addition on the structure and dynamic changes of microbial communities during pickled pepper fermentation. This study is conducive to the establishment of subsequent industrial standards for pickled peppers and provides a theoretical basis for improving their flavor and quality.

## 2 Materials and methods

### 2.1 Preparation and collection of samples

The beer used in the experiment was commercially available Snow Beer (alcohol content ≥3.3 % vol, containing original wort concentration of 9.0°P, malt, beer syrup, and hops). Red chili samples were collected from a chili base, and Erjingtiao chilies with bright red color, no obvious defects, no pest/disease infestation, or physical damage were selected as experimental raw materials. The raw materials were soaked and cleaned with pure water, then air-dried. For all groups, 8 % (*w*/w) salt water was added at a chili-to-salt water ratio of 1:2 (w/w), with no other auxiliary materials added. Experimental groups with different beer addition levels were set: the 25 % beer-added group (25 beer:75 salt water, *v*/v) and the 50 % beer-added group (50 beer:50 salt water, v/v), alongside a control group (KB) without beer addition for fermentation. After thorough mixing, the mixtures were subpackaged into 2 L food-grade glass jars, with 21 jars per group (63 total across the three groups). All samples were fermented at 25 °C for 30 days. Sampling was conducted on days 0, 2, 6, 11, 17, 23, and 30 of fermentation, Three biological replicates were set for each group at each sampling stage (9 jars per time point), and samples were stored frozen in a − 80 °C refrigerator for subsequent analysis.

### 2.2 Determination of physical and chemical indicators

Ten grams of pickled pepper samples were mixed with 90 mL ultrapure water, homogenized, and the pH was measured using a calibrated pH meter (PHS-3C, Shanghai Yidian Scientific Instruments Co., Ltd.); separately, 10 g of pickled pepper samples and 10 g of brine were homogenized for 1 min, filtered, and the filtrate salinity was determined using a digital salinometer (ES-421, ATAGO CO., LTD.) calibrated with standard NaCl solution. Reducing sugar content was assayed via the 3,5-dinitrosalicylic acid method: absorbance was measured at 540 nm, and the content was calculated using a standard curve correlating absorbance with reducing sugar concentration. Total acidity was determined with minor modifications to the automatic potentiometric titration method specified in National Standard GB 12456–2021: 25 g of pickled pepper samples were weighed, homogenized with 50 mL of carbon dioxide-free water, heated in a boiling water bath for 30 min, cooled to room temperature, and then diluted to 250 mL with carbon dioxide-free water; after filtration, 50 mL of the filtrate was rapidly titrated to pH 8.2 ± 0.2 using a 0.1 mol/L NaOH standard titration solution with a fully automatic potentiometric titrator (ZDJ-4B, Shanghai Yidian Scientific Instruments Co., Ltd.), the volume of consumed NaOH standard titration solution was recorded, with water used as a blank control. Nitrite content was determined using a kit (Suzhou Keming Biotechnology Co., Ltd.): under acidic conditions, nitrite reacts with sulfanilic acid to form a diazonium compound, which then couples with N-(1-naphthyl)ethylenediamine to generate a purple-red azo compound, which exhibits a characteristic absorption peak at 540 nm, and the nitrite concentration was calculated by measuring the absorbance and referencing the standard curve. Color difference was measured using an NH300 colorimeter (Shenzhen Senses Time Technology), with the average of ten valid data points taken as the result; for crispness determination, following slight modifications to the method described by Ye et al., 2020, samples were cut into approximately 2 cm × 2 cm cubes and analyzed using a texture analyzer (Shang hai Bosin Tech) with a 36R probe in compression-return mode. The analyzer was operated with a pre-test speed of 1 mm/s, a test speed of 1 mm/s, a post-test speed of 1 mm/s, a compression ratio of 40 %, and a trigger force of 5 g. For each sample, 6 pieces were selected for testing, with each piece tested in triplicate. After excluding data from edge-damaged or abnormal pieces, the average value of 8 valid data points was taken as the final result to reduce the impact of sample heterogeneity.

### 2.3 Sensory evaluation

Sensory evaluation was conducted on pickled pepper samples from the KB group, 25 % beer-added group, and 50 % beer-added group on day 30 of fermentation. The evaluation was completed by 10 experienced sensory assessors majoring in food science. Samples were presented in random order for scoring, with a total of 5 sensory evaluation indicators set: color and texture (20 points), aroma (20 points), acidity (15 points), shape (20 points), and crispness (25 points). Detailed sensory evaluation criteria are shown in Table. S1.

### 2.4 Determination of organic acids

2.0 g of pickled pepper samples were weighed into 50 mL centrifuge tubes, and 18 mL of ultrapure water was added. Ultrasonic extraction was performed for 30 min, followed by centrifugation at 5000 r/min for 10 min. After centrifugation, the supernatant was collected, filtered through a 0.22 μm polytetrafluoroethylene membrane, and analyzed using a high-performance liquid chromatograph (Agilent 1260 Infinity HPLC) equipped with a ZORBAX SB-Aq column (4.6 mm × 250 mm × 5 μm). The chromatographic conditions were as follows: column temperature 37 °C; injection volume 10 μL; flow rate 1 mL/min; mobile phase consisting of 100 % methanol and 0.02 mol/L KH_2_PO_4_ (volume ratio 4:96, pH 2.0); UV detector wavelength 210 nm; and run time 12 min. Organic acids (acetic acid, lactic acid, malic acid, tartaric acid, citric acid, succinic acid, and oxalic acid) were quantified using the external standard method.

### 2.5 Determination of volatile flavor substances

Weigh 2.0 g of the chopped sample into a 20 mL solid-phase microextraction vial， Add 2 mL of saturated brine and 30 μL of 2-octanol (50 μg/mL). The vial was thermally equilibrated at 60 °C on a magnetic stirrer for 10 min, followed by headspace adsorption using a PDMS extraction fiber for 40 min. The fiber was then inserted into the injection port of a gas chromatography-mass spectrometer (GC–MS) maintained at 250 °C for thermal desorption for 5 min. Chromatographic conditions: An HP-5MS capillary column (30 m × 250 μm × 0.25 μm) was used. The carrier gas was high-purity He (99.999 %) at a flow rate of 1 mL/min in splitless mode. The inlet temperature was set at 250 °C. The temperature program was as follows: initial temperature 45 °C, held for 2 min; increased to 85 °C at 3.5 °C/min, held for 2 min; subsequently increased to 126 °C at 2.5 °C/min, held for 2 min; followed by an increase to 140 °C at 1 °C/min; and finally increased to 280 °C at 20 °C/min. Mass spectrometry conditions: Electron ionization (EI) source with 70 eV electron bombardment energy; emission current, 34.6 μA; ion source temperature, 230 °C; quadrupole temperature, 150 °C; interface temperature, 280 °C. The mass scanning range was 20–550 amu. Compounds were identified by retention index (RI) against normal alkanes (C7–C40) and the National Institute of Standards and Technology 17 (NIST 17) database, with a similarity score > 80 %. Relative concentrations of volatiles in pickled peppers were calculated using the following equation:

$$C_{i}=\frac{30\times C_{\text{is}}\times A_{i}}{2.0\times A_{\text{is}.}}$$

In the formula, C_*i*_ represents the mass concentration of any component (μg/kg); C_*is*_ is the mass concentration of the internal standard (μg/mL); A_*i*_ is the chromatographic peak area of any component; A_*is*_ represents the chromatographic peak area of the internal standard, 30 is the volume of the internal standard substance (μL), and 2.0 is the sample mass (g).

### 2.6 Microbial DNA extraction and sequencing

Total DNA from pickled pepper samples was extracted using the FastDNA® SPIN Kit for Soil (MP Biomedicals, Santa Ana, CA). DNA integrity was assessed by agarose gel electrophoresis, and genomic DNA concentration was standardized to 10 ng/μL with total yields exceeding 500 ng using a Qubit 3.0 fluorometer. For bacterial community amplification, the V3–V4 hypervariable region was amplified with primers 341F (CCTACGGGNGGCWGCAG) and 805R (GACTACHVGGGTATCTAATCC); for fungal communities, primers ITS3 (5′-GCATCGATGAAGAACGCAGC-3′) and ITS4 (5′-TCCTCCGCTTATTGATATGC-3′) were used. PCR amplification reactions contained 10 ng/μL sample DNA and spike-in reference DNA in the same system, with positive controls included. Amplified products were initially verified by agarose gel electrophoresis, then subjected to a second PCR using index-containing primers to introduce Illumina-compatible barcodes at the library ends. Sample libraries with unique index tags were appropriately diluted, accurately quantified using Qubit, mixed at the corresponding molar ratios, and purified via gel extraction. The ABI 3730 system was used to confirm the absence of non-specific amplification between 120 and 200 bp and to precisely quantify library concentrations. Sequencing was performed on the Illumina NovaSeq platform (Illumina, San Diego, CA, USA). Relative and absolute quantification of microorganisms was conducted by Genesky Biotechnologies Inc. (Shanghai, China).

### 2.7 Bioinformatics analysis

Raw sequencing data were processed using the Cutadapt plugin in QIIME2 to remove adapters and primers. The DADA2 plugin was then used for quality control and identification of amplicon sequence variants (ASV), including quality filtering, denoising, read merging, and chimera removal. Feature tables and representative sequences were generated. Spike-in sequences were identified, and standard curves for each sample were constructed using read counts. Absolute ASV copy numbers in pickled pepper samples were calculated based on read counts and spike-in copy numbers.

### 2.8 Statistical analysis

All indicators were measured in triplicate, with results expressed as mean ± standard deviation. All data analyses were performed using SPSS 26.0 software. The statistical significance of intergroup differences was assessed via one-way analysis of variance (ANOVA) coupled with Duncan's multiple range test, where *P* < 0.05 was considered statistically significant. Principal component analysis was conducted using SIMCA 14.1 software. Line graphs and bar charts were generated using GraphPad Prism 9.5 software. Microbial interaction networks were constructed in R software (v.4.4.1). Spearman's correlation coefficients were calculated using the OmicShare platform (https://www.omicshare.com/), with |r| > 0.7 and *P* < 0.05 defined as the thresholds for significant correlations. Correlations between dominant microorganisms and key volatile compounds were established, and visualization of these correlation networks was performed using Cytoscape and Gephi software.

## 3 Results and analysis

### 3.1 Sensory evaluation

As shown in Table. 1, the three groups of pickled peppers exhibited significant differences in all sensory indicators (color and texture, aroma, acidity, shape, crispness) and total scores on day 30 of fermentation. The 25 % beer-added group performed better in terms of sensory attributes, with significantly better performance than the other groups in crispness (20.40 ± 1.51), shape (16.40 ± 1.17) and aroma (17.00 ± 1.63). This indicates that the 25 % beer-added has obvious advantages in improving the sensory characteristics of pickled peppers. The 50 % beer-added group performed better in color and texture (17.20 ± 1.03) and acidity (12.80 ± 1.54). In contrast, the pickled peppers in the KB group showed weaker sensory results, especially in aroma and crispness, which further indicates that the addition of 25 % beer can obtain better sensory quality.

**Table 1:** Sensory evaluation scores in three groups of pickled peppers on days 30.

Sensory evaluation score(points)	Sample		
	KB30 d	25 %30 d	50 %30 d
Color and lustre (20)	14.90 ± 2.47^b^	16.50 ± 1.27^a^	17.20 ± 1.03^a^
Aroma (20)	15.60 ± 0.97^b^	17.00 ± 1.63^a^	15.90 ± 1.29^ab^
Acidity (15)	10.50 ± 2.07^b^	12.60 ± 1.51^a^	12.80 ± 1.54^a^
Shape (20)	12.30 ± 1.64^c^	16.40 ± 1.17^a^	14.60 ± 1.71^b^
Texture (25)	16.50 ± 1.35^c^	20.40 ± 1.51^a^	18.40 ± 1.58^b^
In total	69.80 ± 2.49^c^	82.90 ± 2.92^a^	78.90 ± 4.82^b^

Note: Data represent mean ± standard deviation. Different lowercase letters (a-b) indicate significant differences among the three groups of pickled peppers for the same parameter (*P* < 0.05, Confidence Interval:CI95%). KB30 d represents the sample of pickled peppers from the KB group fermented at 30 days of fermentation; 25 % 30d represents the sample with 25 % beer-added fermented at 30 days of fermentation; and 50 %30d represents the sample with 50 % beer-added fermented at 30 days of fermentation.

### 3.2 Physicochemical property analysis

**Fig. 1:**
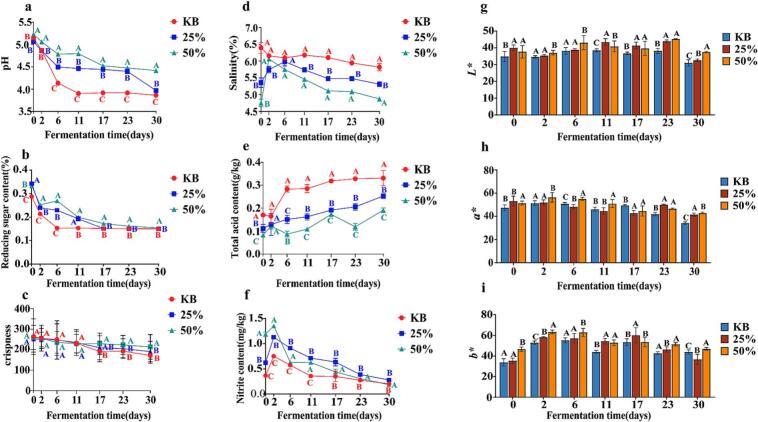
Physicochemical indicators during pickled pepper fermentation: (a) pH, (b) reducing sugar, (c) crispness, (d) salinity, (e) total acid, (f) nitrite, (g) L*, (h) a*, and (i) b*. Note. Letters A-C represent the significance analysis among the three pickled pepper groups at the same fermentation stage (*P* < 0.05, Confidence Interval:CI95%).

Fig. 1 illustrates the temporal dynamics of key physicochemical parameters during pickled pepper fermentation. As depicted in Fig. 1a, the pH values of the KB group declined from 5.15 to 3.86 during fermentation. In contrast, the 25 % and 50 % beer-added groups exhibited pH ranges of 5.06–3.97 and 5.23–4.42, respectively. Conversely, the total acid content (Fig. 1e) increased steadily with the progression of fermentation. After 30 days of fermentation, the KB group had a total acid content of 0.33 g/kg, significantly higher than that of the 25 % beer-added group (0.25 g/kg) and the 50 % beer-added group (0.19 g/kg). This phenomenon is attributed to the metabolic activities of lactic acid bacteria and other microorganisms, which produce organic acids during fermentation. These acids lower the system pH and contribute to the accumulation of total acid (Yang, Luo, et al., 2021). As shown in Fig. 1f, the nitrite content initially increased and subsequently decreased over the fermentation period. Specifically, the KB group showed nitrite fluctuations between 0.20 and 0.75 mg/kg, compared to 0.28–1.13 mg/kg in the 25 % beer group and 0.19–1.35 mg/kg in the 50 % beer group. Notably, all measured values remained well below the 20 mg/kg safety threshold stipulated by China's National Food Safety Standard (GB 2762–2022). The nitrite levels in the 25 % and 50 % beer-added groups were higher than those in the KB group. This discrepancy might be attributed to the lower pH and more active lactic acid bacteria in the KB group, which inhibited nitrate reductase activity and retarded nitrite formation (Zhao et al., 2021). As shown in Fig. 1d, salinity decreased with fluctuations throughout the fermentation process, reaching final values of 5.82 % (KB), 5.31 % (25 %), and 4.87 % (50 %) after 30 days. The reducing sugar content in the beer-added groups (25 % and 50 %) remained consistently higher than that in the KB group throughout fermentation (Fig. 1b). However, as microbial metabolic activity intensified, reducing sugars in all groups were continuously consumed, exhibiting a sustained downward trend that eventually plateaued. Crispness is a core indicator for evaluating the quality of pickled peppers and one of the key factors affecting consumer acceptance. As shown in Fig. 1c, the crispness of pickled peppers in all three groups showed a decreasing trend during fermentation: the KB group decreased from 263.04 to 171.37, the 25 % beer-added group from 259.72 to 213.54, and the 50 % beer-added group from 252.73 to 190.53. These results indicate that beer addition can improve the textural crispness of pickled peppers, with the 25 % beer-added group achieving the optimal effect, which is consistent with the sensory evaluation results. The L*, a*, and b* values in the 25 % and 50 % beer groups (Fig. 1g–i) were significantly higher than those in the KB group, with statistically significant differences among all three groups (*P* < 0.05), indicating that beer addition protected the color of pickled peppers.

### 3.3 Comparative analysis of microorganisms

#### 3.3.1 Analysis of microbial α -diversity

Based on Observed and Chao1 indices (Fig. S1a–d), bacterial richness in the KB group first increased then decreased during fermentation, whereas the 25 % and 50 % beer-added groups showed continuous richness growth, stabilizing in the late stage. At the end of fermentation, both beer-added groups had significantly higher bacterial richness than the KB group. Notably, the fungal richness of the KB group declined continuously, indicating intense resource competition in the natural fermentation community. For fungal richness, the 25 % beer-added group showed a decline-stabilization trend, while the 50 % group decreased initially but increased from day 17 onward, suggesting 50 % beer addition may reconstruct the microniche to promote specific functional fungi via environmental modulation in late fermentation. Shannon and Simpson indices (Fig. S1e–h) showed similar trends for both bacterial and fungal communities, with diversity fluctuating throughout fermentation.

#### 3.3.2 Analysis of microbial β-diversity

Dynamic changes in the β-diversity of bacterial and fungal communities during pickled pepper fermentation were analyzed by principal coordinate analysis (PCoA) and non-metric multidimensional scaling (NMDS) based on Bray-Curtis distances. PCoA of bacterial communities (Fig. S2a) revealed that the unfermented stage (0 d) of the three pickled pepper groups showed distinct separation and was distant from other stages. This was attributed to the distinct bacterial communities harbored by raw pepper materials at the unfermented stage, leading to low similarity in bacterial community structure and large distances between samples. All bacterial PCoA samples exhibited a counterclockwise shift during fermentation, with samples at 23 and 30 days of fermentation showing a clustering trend. PCoA of fungal communities (Fig. S2c) indicated clear separation of samples across different fermentation stages. The Stress values for bacterial (Fig. S2b) and fungal (Fig. S2d) NMDS analyses were 0.11 and 0.18, respectively. Thus, the results of this study are reliable.

#### 3.3.3 Comparison of bacterial community composition

In this study, absolute and relative quantification methods were used to analyze the bacterial community structure and its dynamic changes during the fermentation of pickled peppers with beer addition. as shown in Fig. S3a and 3b, a total of 2 dominant bacterial phyla were detected in the three groups of pickled peppers, namely Firmicutes and Proteobacteria (average relative abundance >1 %). At the unfermented stage (0 d), the dominant phylum in the KB group was Proteobacteria, while both the 25 % beer-added group and 50 % beer-added group had Firmicutes and Proteobacteria as the dominant phyla. From day 2 to day 30 of fermentation, Firmicutes and Proteobacteria remained the dominant phyla in all three groups of pickled peppers, but their succession trends varied. In the KB group, the absolute abundance of Firmicutes first increased and then decreased, whereas that of Proteobacteria first decreased and then increased. In contrast, in the 25 % beer-added group and 50 % beer-added group, the absolute abundance of Firmicutes first decreased and then increased, while that of Proteobacteria first increased and then decreased. Firmicutes and Proteobacteria are the most abundant bacterial phyla in fermented peppers (Wang et al., 2019), which further indicates that pickled peppers produced by different fermentation techniques share similarities in their bacterial community structure at the phylum level.

**Fig. 2:**
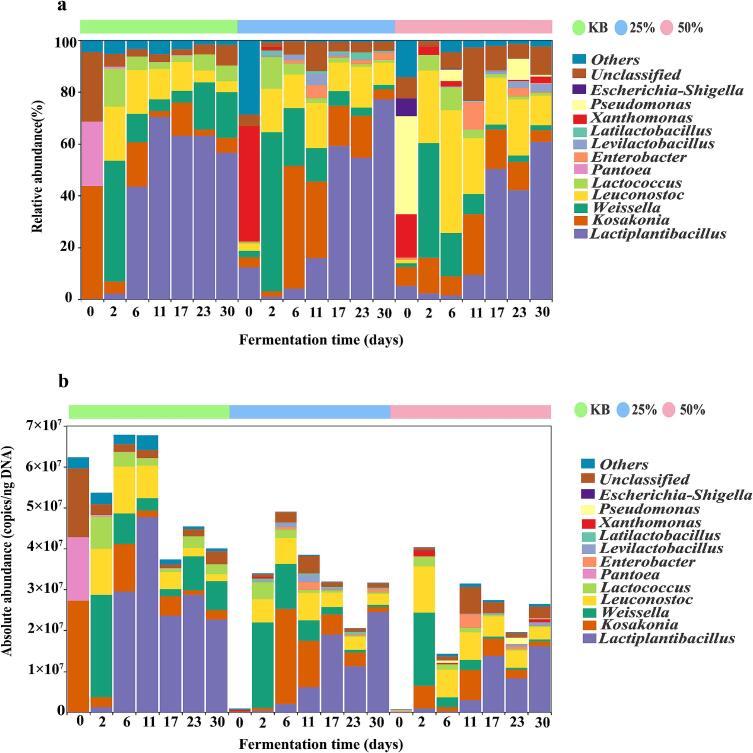
Composition of bacterial communities in three pickled pepper groups. (a) Relative abundance of bacteria at the genus level; (b) Absolute abundance of bacteria at the genus level.

At the bacterial genus level, the KB group, 25 % beer-added group, and 50 % beer-added group contained 6, 9, and 10 dominant genera (average relative abundance >1 %), respectively; as shown in Fig. 2a and b, shared dominant genera across all three groups were *Lactiplantibacillus*, *Kosakonia*, *Weissella*, *Leuconostoc*, and *Lactococcus*. At the unfermented stage (0 d), the KB group was dominated by *Kosakonia* and *Pantoea*, the 25 % beer-added group by *Xanthomonas* and *Lactiplantibacillus*, and the 50 % group by *Pseudomonas* and *Xanthomonas*. As fermentation progressed, *Lactiplantibacillus* gradually became dominant in all groups but exhibited distinct dynamics, in the KB group, its absolute abundance first increased and then decreased, peaking on day 11 (4.77 × 10^7^ copies/ngDNA) and declining to 2.27 × 10^7^ copies/ngDNA by day 30, while in the 25 % and 50 % beer-added groups, its absolute abundance gradually increased, both reaching peaks on day 30 at 2.45 × 10^7^ copies/ngDNA and 1.61 × 10^7^ copies/ngDNA, respectively. By day 30 of fermentation, the 25 % beer-added group showed a slightly higher absolute abundance of *Lactiplantibacillus* compared to the other two groups. Additionally, the growth of *Weissella* was inhibited by *Lactiplantibacillus*, which is consistent with the findings of. Wang et al., 2019. The absolute abundance of *Weissella* in all three groups peaked on day 2 of fermentation, at 2.51 × 10^7^ copies/ngDNA (KB), 2.09 × 10^7^ copies/ngDNA (25 %), and 1.79 × 10^7^ copies/ngDNA (50 %), respectively. on day 30 of fermentation, their absolute abundances had decreased significantly to 7.09 × 10^6^ copies/ngDNA (KB), 5.43 × 10^5^ copies/ngDNA (25 %), and 5.10 × 10^5^ copies/ngDNA (50 %), respectively, indicating that *Weissella* plays an active role in the early stage of pickled pepper fermentation. *Weissella* and *Lactiplantibacillus* can produce various volatile flavor substances such as alcohols, esters, and acids through metabolism, which synergistically contribute to the formation of the characteristic sensory quality of pickled peppers (Yang, Xia, et al., 2021). Compared with previous studies, *Rosenbergiella*, *Staphylococcus*, *Lactobacillus*, and *Pseudomonas* have been reported as dominant bacterial genera in fermented peppers (Cai et al., 2021; Xu et al., 2020; Xu, Chen, et al., 2021), which differs from the results of this study; this discrepancy may be attributed to the influence of fermentation substrates, environmental conditions, and process parameters on the microbial community (Liu et al., 2023).

#### 3.3.4 Comparison of fungal community composition

The community succession at the fungal phylum level is shown in Fig. S4a and 4b. The dominant fungal phyla (average relative abundance >1 %) in both the KB group and the 50 % beer-added group were Ascomycota and Fungi_phy_Incertae_sedis, while the 25 % beer-added group exhibited a more diverse community structure, including Ascomycota, Fungi_phy_Incertae_sedis, and Basidiomycota. During the 0–30 d fermentation period, Ascomycota gradually became the absolutely dominant phylum in all three groups of pickled peppers. Specifically, the absolute abundances of Ascomycota and Fungi_phy_Incertae_sedis in the KB group increased gradually with fermentation time. The absolute abundances of Ascomycota, Fungi_phy_Incertae_sedis, and Basidiomycota in the 25 % beer-added group showed fluctuating changes. in the 50 % beer-added group, the absolute abundance of Ascomycota exhibited a trend of first increasing and then decreasing, while the absolute abundance of Fungi_phy_Incertae_sedis decreased gradually with fermentation time.

**Fig. 3:**
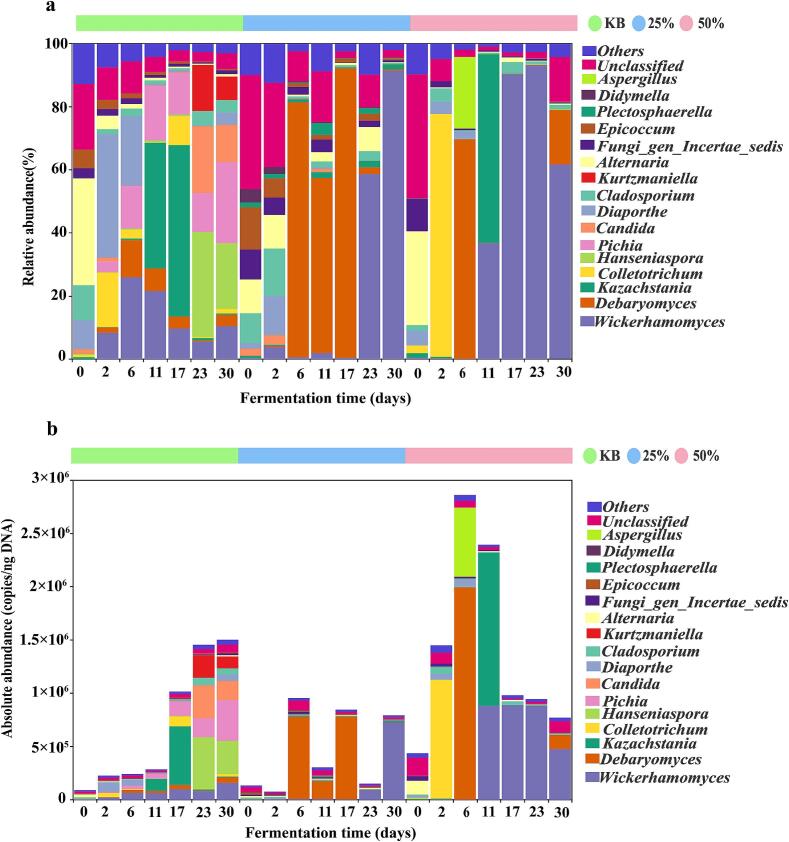
Composition of fungal communities in three pickled pepper groups. (a) Relative abundance of fungi at the genus level; (b) Absolute abundance of fungi at the genus level.

In addition to bacteria, fungi also play a critical role in flavor development during pickled pepper fermentation. as shown in Fig. 3a and b. The three groups share dominant fungal genera, including *Wickerhamomyces*, *Debaryomyces*, *Kazachstania*, *Diaporthe*, *Cladosporium*, *Alternaria*, and *Fungi_gen_Incertae_sedis*, though significant differences exist in their community succession patterns. In the KB group, during 0–6 d of fermentation, the absolute abundances of *Wickerhamomyces* and *Debaryomyces* increased from undetectable levels at 0 d to 6.24 × 10^4^ copies/ng DNA and 2.84 × 10^4^ copies/ng DNA at 6 d, respectively, while those of *Alternaria* and *Epicoccum* gradually decreased. A shift in dominant genera occurred during 11–17 d: the absolute abundance of *Kazachstania* increased from 1.13 × 10^5^ copies/ng DNA at 11 d to 5.52 × 10^5^ copies/ng DNA at 17 d, becoming the dominant genus. During this stage, *Colletotrichum* also proliferated significantly, with its absolute abundance rising from 1.90 × 10^2^ copies/ng DNA at 11 d to 9.35 × 10^4^ copies/ng DNA at 17 d. In the late fermentation period (23–30 d), the absolute abundances of *Wickerhamomyces* (1.57 × 10^5^ copies/ng DNA), *Debaryomyces* (5.38 × 10^4^ copies/ng DNA), and *Pichia* (3.85 × 10^5^ copies/ng DNA) all reached their peaks at 30 d. Notably, the highly active polygalacturonase produced by *Pichia* may lead to reduced hardness and tissue damage in fermented vegetables (Xu, Wu, et al., 2021). Meanwhile, potential harmful fungi such as *Colletotrichum* maintained a certain abundance, which could threaten product stability and safety (Yang et al., 2025).

Compared with the KB group, *Wickerhamomyces*, *Debaryomyces*, and *Kazachstania* were the core dominant genera in both the 25 % and 50 % beer-added groups, with distinct dynamic changes: In the 25 % beer-added group, the absolute abundance of *Wickerhamomyces* increased continuously, gradually rising from 3.45 × 10^2^ copies/ng DNA at 0 d to 7.23 × 10^5^ copies/ng DNA at 30 d. *Debaryomyces* showed a trend of first increasing and then decreasing, peaking at 7.76 × 10^5^ copies/ng DNA at 17 d before declining to 1.18 × 10^3^ copies/ng DNA at 30 d. The abundance of *Kazachstania* fluctuated slightly, reaching a peak of 1.32 × 10^4^ copies/ng DNA at 30 d. In the 50 % beer-added group, the absolute abundances of *Wickerhamomyces* and *Kazachstania* both peaked at 11 d, rising from 1.40 × 10^3^ copies/ng DNA and 6.34 × 10^3^ copies/ng DNA at 0 d to 8.81 × 10^5^ copies/ng DNA and 1.43 × 10^6^ copies/ng DNA at 11 d, respectively, before decreasing to 4.75 × 10^5^ copies/ng DNA and 1.18 × 10^3^ copies/ng DNA at 30 d. Debaryomyces peaked at 1.99 × 10^6^ copies/ng DNA at 6 d of fermentation, then decreased rapidly to 1.18 × 10^3^ copies/ng DNA at 30 d. Additionally, the 25 % beer-added group showed no obvious proliferation of *Colletotrichum*, whereas in the 50 % beer-added group, *Colletotrichum* dominated at 2 d of fermentation with an absolute abundance of 1.12 × 10^6^ copies/ng DNA, then decreased continuously to 1.61 × 10^3^ copies/ng DNA at 30 d, which was lower than the absolute abundance in the KB group during the same period. Beer addition significantly enhanced the ecological competitive advantage of *Wickerhamomyces* and *Debaryomyces* in the pickled pepper fermentation system, likely because the rich nutrients in beer (e.g., sugars and amino acids) provide a suitable growth substrate for these genera (Canonico et al., 2019; Loman et al., 2018). Among them, *Debaryomyces* improves the quality and aroma of fermented peppers by promoting the synthesis of alcohols, aldehydes, and esters (Zhao et al., 2016). *Wickerhamomyces* can secrete proteases that hydrolyze proteins into small peptides and amino acids, which serve as flavor precursors, contributing to umami taste and enhancing the sensory properties of fermented foods (Zha et al., 2018). *Kazachstania* can ferment sugars to produce high levels of acetate esters, thereby promoting flavor formation (Lin et al., 2023). This is consistent with the detection results of volatile flavor compounds: the contents of alcohols, aldehydes, and esters in the beer-added groups (25 % and 50 %) were all higher than those in the KB group, indicating that beer addition can promote the production of volatile flavor compounds in pickled peppers by enriching functional microorganisms. Compared with previous studies, Xu, Chen, et al., 2021 reported that the fungal community in fermented peppers is dominated by *Hyphopichia* and *Kodamaea*, a finding that differs from the results of this study. This discrepancy may be due to the rich nutrients in beer providing favorable growth conditions for the microbial community, and its mechanism of action requires further verification. Huang, Li, Wu, Guo, et al., 2025 found that *Wickerhamomyces* dominates during the fermentation of Zhuzijiao, which aligns with the dominant status of this genus in this study, indicating that it may have strong environmental adaptability in pepper fermentation. Beer addition altered the fungal community structure of the system, enriching key functional microorganisms while effectively inhibiting the proliferation of pathogenic microorganisms. Under the conditions of this study, The 25 % beer-added group exhibited favorable fermentation potential.

### 3.4 Analysis of microbial co-occurrence networks

**Fig. 4:**
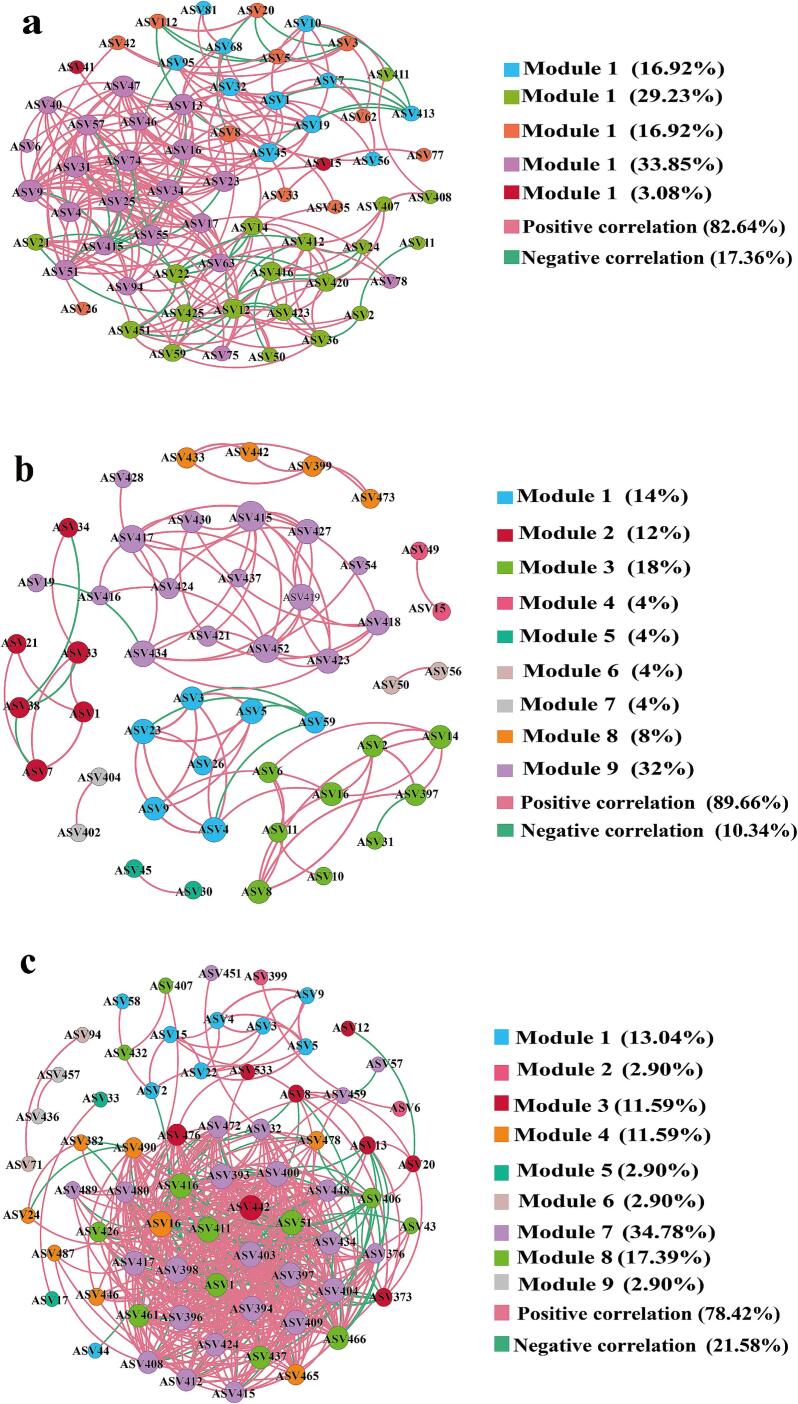
Microbial interaction network diagram of three groups of pickled peppers. (a) KB group; (b)25 % beer-added group; (c) 50 % beer-added group. Note: Each node represents an ASV, where the node size corresponds to the degree of correlation with other microbial genera. Blue edges denote positive correlations, while orange edges indicate negative correlations. (For interpretation of the references to color in this figure legend, the reader is referred to the web version of this article.)

Co-occurrence networks constructed using Spearman correlation coefficients (|r| > 0.7, *p* < 0.05) revealed microbial interactions under different fermentation conditions. As depicted in Fig. 4a-c, the KB group network comprised 65 nodes and 265 edges. The 25 % beer-added group exhibited reduced complexity (20 nodes, 87 edges), whereas the 50 % beer-added group showed a significant increase (69 nodes, 519 edges), forming a densely interconnected structure. Network metrics in the 50 % beer-added group increased significantly: average degree from 8.154 (KB) to 15.034, density from 0.127 to 0.221, and average clustering coefficient from 0.634 to 0.734. Conversely, average path lengths decreased from 4.234 (KB) to 2.302 (25 %) and 2.581 (50 %). Shorter average path lengths indicate more efficient information, energy, and matter transfer among microbial species. The 25 % beer-added group exhibited a significantly higher proportion of positive correlations than the KB group. In fermented food production, positive microbial interactions are critical for enhancing substrate utilization and process efficiency (Wang et al., 2023).

### 3.5 Organic acid analysis

Organic acids can reduce bitterness, enrich taste layers, and enhance aroma. As shown in Fig. S5, the 25 % and 50 % beer-added groups mainly contained Lactic and Citric acids, while the KB group was dominated by Lactic and Succinic acids. Oxalic, Tartaric, Malic, and Acetic acids were present in low amounts with fluctuating changes in all samples. At the unfermented stage (0 d), the KB group had a significantly higher lactic acid content (2.24 mg/g) than the 25 % (0.79 mg/g) and 50 % (1.45 mg/g) beer-added groups. on day 30 of fermentation, Lactic acid increased significantly in all groups, reaching 18.30 mg/g (KB), 13.86 mg/g (25 %), and 12.41 mg/g (50 %). The lactic acid content in beer-added groups was consistently lower than that in the KB group, with the 50 % beer-added group showing the lowest total accumulation, which may be associated with the inhibitory effect of components such as ethanol and hops in beer on certain lactic acid bacteria (Vriesekoop et al., 2012). Citric acid showed a fluctuating downward trend in all groups, and the 25 % and 50 % beer-added groups consistently had higher levels than the KB group. As an antioxidant, Citric acid can mitigate color changes caused by browning oxidation (Kim et al., 2023), thus, the higher Citric acid content in beer-added groups may be more conducive to maintaining the color stability of pickled peppers. Succinic acid fluctuated in all groups. on day 30 of fermentation, the succinic acid contents in the KB group, 25 % beer-added group, and 50 % beer-added group were 0.93 mg/g, 0.89 mg/g, and 1.21 mg/g, respectively. Succinic acid imparts bitter, salty, and sour tastes, and its lower content helps improve sensory quality (Vilela, 2019).

### 3.6 Analysis of volatile flavor compounds

#### 3.6.1 Dynamic analysis of volatile flavors

**Fig. 5:**
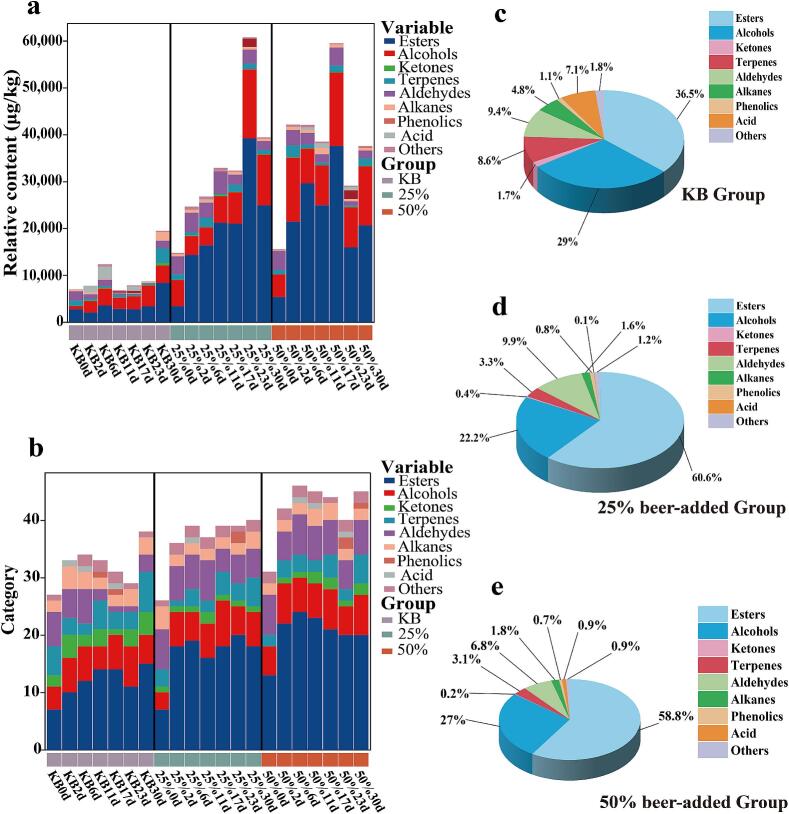
Volatile flavor compounds during the fermentation of three groups of pickled peppers. (a) The content of each type of volatile flavor compound; (b) The number of volatile flavor compounds; (c) Relative percentage contents of various volatile flavor compounds in the KB group; (d) Relative percentage contents of various volatile flavor compounds in the 25 % beer-added group; (e) Relative percentage contents of various volatile flavor compounds in the 50 % beer-added group.

Volatile flavor compounds in the KB, 25 % beer-added, and 50 % beer-added groups were detected via HS-SPME-GC–MS. Table. S2–4 show 81 volatile compounds identified across the three samples, including 31 esters, 15 alcohols, 8 ketones, 9 terpenes, 9 aldehydes, 4 alkanes, 2 phenols, 1 acid, and 2 others. Fig. 5a–b indicate that beer addition significantly promoted the production and accumulation of volatile flavor substances in pickled peppers. As shown in Fig. 5c–e, esters accounted for the highest relative content (36.5–60.6 %) among the three groups, followed by alcohols (22.2–29 %), aldehydes (6.8–9.9 %), and terpenes (3.1–8.6 %). Esters are key aromatic compounds imparting typical fruity and floral notes to pickled peppers (Chen et al., 2024); Ye et al., 2020 also confirmed that esters are the main volatile components in fermented peppers. Compared with the KB group, the 25 % beer-added group had 7 new esters (e.g., ethyl benzoate, ethyl caprate, methyl linoleate, ethyl undecanoate), and the 50 % beer-added group had 9 new esters (e.g., methyl tetradecanoate, methyl stearate, ethyl laurate, methyl decanoate). Previous studies note that ethyl benzoate, ethyl caprate, and methyl decanoate provide wintergreen oil-like, fruity, floral, and alcoholic flavors, respectively (Chen et al., 2024; Li, Wang, et al., 2023). Detected esters were mainly ethyl esters, with ethyl acetate, elaidic acid ethyl ester, and ethyl linoleate present at higher levels in beer-added groups. Ethyl linoleate was only detected in the KB group on days 0 and 30 (46.20 μg/kg and 389.08 μg/kg, respectively), while in 25 % and 50 % beer-added groups, it showed a trend of first increasing and then decreasing, reaching 3376.03 μg/kg and 3201.28 μg/kg on day 30. ethyl linoleate has physiological functions such as enhancing immunity and reducing cholesterol and blood lipids (Koo et al., 2014), and its high accumulation in beer-added groups endows these pickled peppers with potential health-promoting value.

As the second most abundant volatile component, total alcohol content increased with beer addition: 20162.76 μg/kg (KB), 51290.13 μg/kg (25 %), and 71,223.07 μg/kg (50 %), indicating significant promotion of alcohol accumulation by beer. Compared with the KB group, isoamyl alcohol and nerolidol were detected in beer-added groups; these are typical higher alcohol flavor substances in beer (Xie et al., 2021), suggesting they may have been introduced via beer. Phenethyl alcohol was only detected in the KB group on day 30 (1590.94 μg/kg), while in 25 % and 50 % beer-added groups, it showed a trend of first increasing and then decreasing, reaching 4322.96 μg/kg and 6283.53 μg/kg on day 30. phenethyl alcohol mainly derives from phenylalanine catabolism during yeast fermentation and plant secondary metabolism (Holt et al., 2019), it is hypothesized that beer addition may enhance the phenylalanine catabolic pathway, thereby increasing its content.

Aldehydes and terpenes also affect pickled pepper flavor. Aldehydes, mainly from fatty acid oxidation and amino acid degradation, impart fresh or fruity notes (Zhang, He, et al., 2023). For example, phenylacetaldehyde has typical floral and sweet flavors (Wang et al., 2017), its total content in 25 % (1520.46 μg/kg) and 50 % (1508.88 μg/kg) beer-added groups was 3.67 and 3.64 times higher than in the KB group (414.32 μg/kg), indicating significant promotion by beer. Terpenes contribute to woody, floral, and sweet notes (Ye et al., 2020); common ones include d-limonene, β-elemene, (+)-valencene, phenylethylene. β-elemene, with a fresh cucumber-like odor, is an important aromatic compound widely present in peppers (Sun et al., 2020).

#### 3.6.2 Analysis of differential volatile flavor substances

**Fig. 6:**
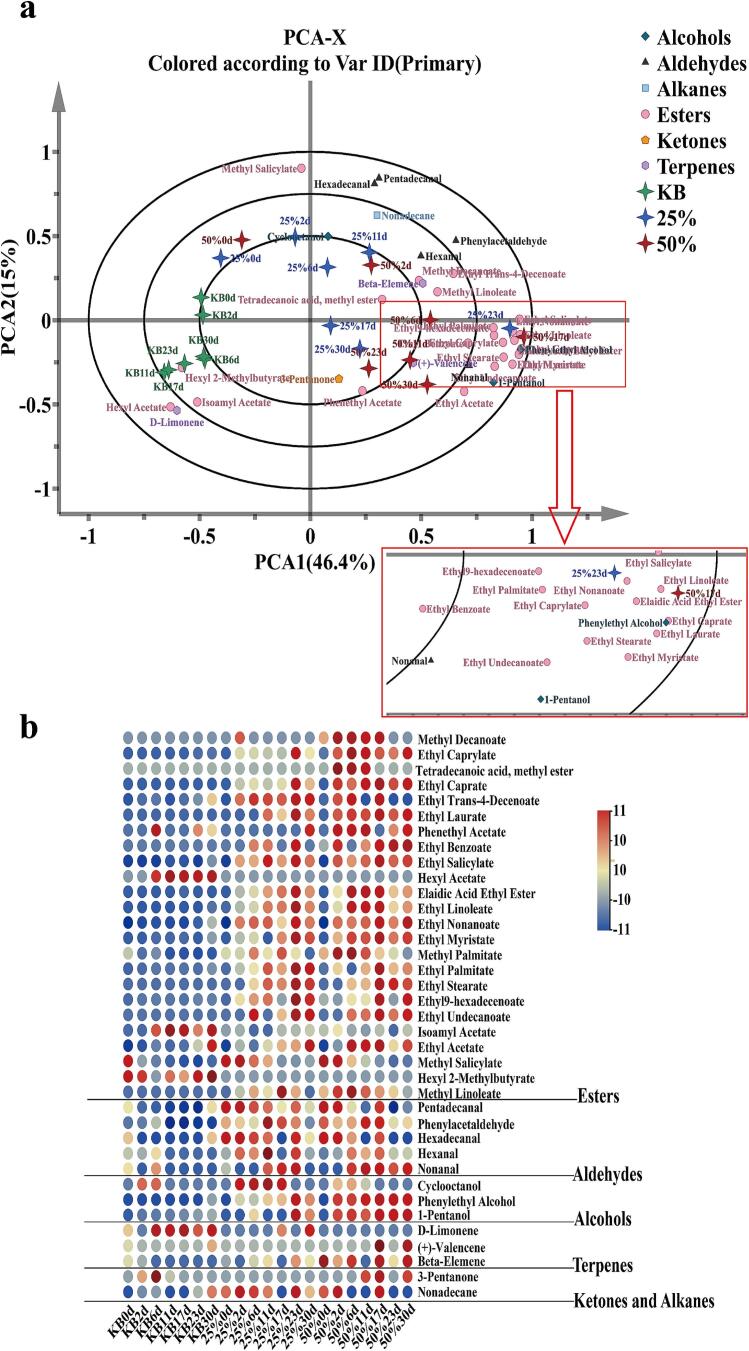
Analysis of differentially volatile flavor compounds in three pickled pepper groups. (a) PCA-Biplot of differentially volatile flavor compounds; (b) Cluster heatmap of differentially volatile flavor compounds. Note: The content in the large red box at the bottom of Fig. 6a is identical to that in the small red box of the Biplot, with only magnification applied. (For interpretation of the references to color in this figure legend, the reader is referred to the web version of this article.)

To investigate the differential volatile flavor compounds among three groups of pickled peppers, a total of 37 differential volatile compounds were identified in this study with the screening criteria of VIP ≥ 1 and *P* < 0.05. These compounds comprised 24 esters, 5 aldehydes, 3 alcohols, 3 terpenes, 1 acid, and 1 ketone. PCA-Biplot analysis was performed based on these 37 differential volatile compounds, where the spatial distance between sample points and volatile substances could effectively characterize the degree of their correlation. As shown in Fig. 6a and b, the variance contribution rates of PCA1 and PCA2 were 46.4 % and 15 %, respectively, with a cumulative contribution rate of 61.4 %, indicating that the first two principal components could effectively account for the main differences in the volatile flavors of the samples. Sample points of the KB group were concentrated on the left half-axis of PCA1, while those of the 25 % beer-added group and 50 % beer-added group were mainly distributed on the right half-axis of PCA1. Notably, the sample points of the latter two groups were spatially close to each other but far from those of the KB group, suggesting that the volatile flavor compounds of the 25 % beer-added group and 50 % beer-added group were more similar, whereas they differed from those of the KB group. Specifically, the highest abundance of flavor substances, particularly esters, was observed around day 23 day in the 25 % beer-added group and day 17 in the 50 % beer-added group, which corresponded to the peak periods of ester synthesis in the two groups of pickled peppers, respectively. The main characteristic esters included 16 ester compounds such as ethyl caprylate, ethyl nonanoate, and ethyl salicylate. Flavor substances close to the KB group included d-limonene, hexyl acetate, isoamyl acetate, and hexyl 2-methylbutyrate, among which hexyl acetate and hexyl 2-methylbutyrate were unique to the KB group and exhibited dynamic accumulation in their contents. The content of hexyl 2-methylbutyrate reached a maximum of 79.32 μg/kg on day 30, while hexyl acetate was detected from day 6, increased to 596.05 μg/kg on day 11, then decreased slightly, and rebounded to 487.41 μg/kg on day 30. The specific accumulation of these two compounds collectively constituted the differential volatile compounds that distinguished the KB group from the beer-added groups.

### 3.7 Correlation analysis of dominant microorganisms and differential volatile flavors

**Fig. 7:**
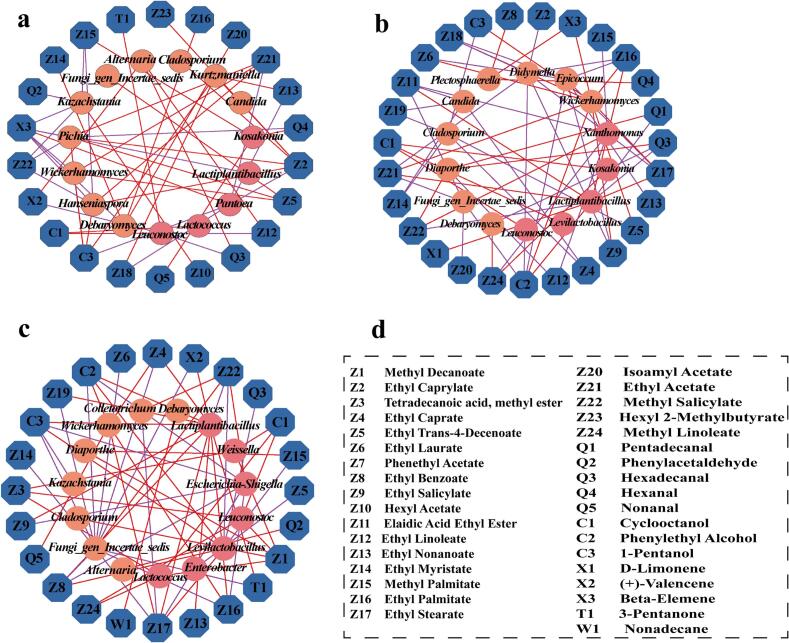
Correlation network diagrams of dominant bacterial genera and characteristic volatile flavor compounds in three pickled pepper groups. (a) KB group; (b)25 % beer-added group; (c) 50 % beer-added group. Note: The inner pink and orange circles represent bacteria and fungi, respectively, while the outer blue circle denotes differentially volatile flavor compounds. Red and purple edges signify significant positive (*r* > 0.7, *P* < 0.05) and negative (*r* < −0.7, *P* < 0.05) correlations, respectively. (For interpretation of the references to color in this figure legend, the reader is referred to the web version of this article.)

Correlation analysis was performed between dominant microorganisms (average relative abundance >1 %) and 37 differential volatile flavor substances, based on Spearman correlation coefficients (|r| > 0.7, *P* < 0.05) (Fig. 7a–c). The results revealed 53 significant correlations in the KB group (32 positive, 21 negative), 66 significant correlations in the 25 % beer-added group (28 positive, 38 negative), and 64 significant correlations in the 50 % beer-added group (38 positive, 26 negative). In the KB group, *Pichia* and *Pantoea* were the genera most closely associated with flavor substances; specifically, *Pichia* showed significant positive correlations with ethyl acetate, ethyl caprylate, ethyl trans-4-decenoate, and 1-pentanol, while exhibiting negative correlations with methyl salicylate, hexanal, and beta-elemene. It has been reported that *Pichia* can produce small amounts of flavor compounds such as acids and alcohols, which exert a moderate promoting effect on the flavor of pickled peppers (Zhao et al., 2016). In contrast, *Pantoea* primarily displayed negative correlations, including those with ethyl caprylate, ethyl nonanoate, ethyl 9-hexadecenoate, ethyl acetate, and 1-pentanol, with only a positive correlation observed with beta-elemene. Notably, *Lactiplantibacillus* and *Wickerhamomyces* emerged as the core microorganisms associated with flavor substances in the beer-added groups: in the 25 % beer-added group, *Lactiplantibacillus* was positively correlated with 6 compounds (4 esters, 1 alkene, and 1 alcohol), whereas in the 50 % beer-added group, it showed positive correlations with 10 compounds (7 esters, 2 alcohols, and 1 ketone). *Lactiplantibacillus* can promote ester synthesis, endowing fermented peppers with fruity and sweet notes and enhancing the overall flavor (Yang et al., 2025). For *Wickerhamomyces*, in the 25 % beer-added group, it exhibited significant positive correlations with 10 flavor substances (7 esters, 2 alcohols, and 1 alkene), while in the 50 % beer-added group, it was positively correlated with 5 flavor compounds (3 esters, 1 alcohol, and 1 aldehyde); *Wickerhamomyces* metabolizes carbohydrates and proteins to produce aldehydes, alcohols, and esters, thereby enhancing the flavor of fermented foods (Huang, Li, Wu, Tong, et al., 2025).

### 3.8 Construction of metabolic pathways

**Fig. 8:**
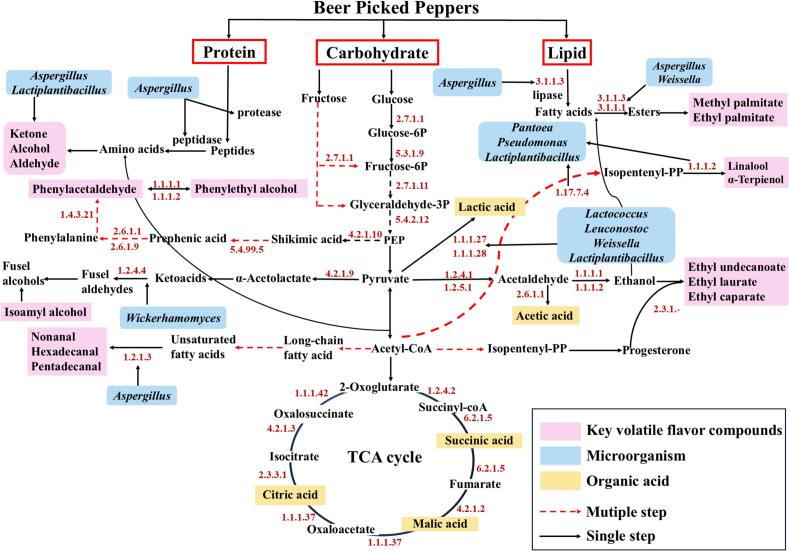
Prediction of metabolic pathways for key flavor compounds in beer pickled peppers. Note: Pink denotes key volatile flavor compounds, blue represents microorganisms, yellow indicates organic acids, red dashed lines stand for multiple steps, and black solid lines signify single steps. (For interpretation of the references to color in this figure legend, the reader is referred to the web version of this article.)

Based on the KEGG database and related literature, this study constructed metabolic pathways for flavor compounds in beer pickled peppers (Fig. 8) and predicted the activities of relevant enzymes secreted by microorganisms in the three groups of pickled peppers using PICRUSt2 (Fig. S6). As shown in Fig. 8, after the three major nutrients in peppers, carbohydrates, proteins, and lipids, are initially degraded into sugars, amino acids, and fatty acids, they can generate corresponding flavor substances through specific metabolic pathways under the catalysis of different functional enzymes. In terms of protein metabolism, proteins are decomposed into small-molecule peptides by proteases secreted by *Aspergillus*, which are further hydrolyzed into amino acids; under the synergistic action of *Aspergillus* and *Lactiplantibacillus*, these amino acids can combine with acetyl-CoA to produce flavor precursor substances such as ketones, alcohols, and aldehydes. In lipid metabolism, lipids are degraded into fatty acids by lipase (EC: 3.1.1.3); with the participation of carboxylesterase (EC: 3.1.1.1), these fatty acids react with ethanol to form fatty acid esters like methyl palmitate and ethyl palmitate, EC: 3.1.1.1 and EC: 3.1.1.3 can be secreted by *Weissella* and *Aspergillus* (Zhang et al., 2016). Additionally, *Aspergillus* converts long-chain saturated and unsaturated fatty acids into nonanal, hexadecanal, and pentadecanal via aldehyde dehydrogenase (EC 1.2.1.3).

Glucose and fructose are the most important carbon sources in peppers; they are converted into fructose-6P, glyceraldehyde 3-phosphate, and phosphoenolpyruvate (PEP) through glycolysis and fructose metabolism pathways, and participate in various other metabolic reactions, with involved enzymes including EC: 2.7.1.1, EC: 5.3.1.9, EC: 2.7.1.11, and EC: 5.4.2.12. As a key intermediate, pyruvate can be further converted into lactic acid, acetic acid, and acetyl-CoA, and then enter the tricarboxylic acid cycle (TCA cycle). Lactic acid can be generated via EC: 1.1.1.27 and EC: 1.1.1.28, with the main microorganisms involved in this process being *Lactococcus*, *Leuconostoc*, *Weissella*, and *Lactiplantibacillus*. Pyruvate can also be converted into acetaldehyde by EC: 1.2.4.1 and EC: 1.2.5.1; subsequently, acetaldehyde is further converted into ethanol via EC: 1.1.1.1 and EC: 1.1.1.2, thereby promoting the formation of ester compounds such as ethyl caprate, ethyl laurate, and ethyl undecanoate (Li, Luo, et al., 2023). Phenylalanine, as a flavor amino acid and precursor of higher alcohols, is synthesized from glucose through the glycolysis pathway (Li, Wang, et al., 2023), in this study, phenylalanine is converted into phenylacetaldehyde under the catalysis of EC: 1.4.3.21, and phenylacetaldehyde is further converted into phenylethanol via EC: 1.1.1.1 and EC: 1.1.1.2 (Zhao et al., 2022). Moreover, linalool and α-terpineol are mainly produced through the terpene biosynthesis pathway of *Pantoea*, *Pseudomonas*, and *Lactiplantibacillus*.

## 4 Conclusion

This study investigated the effects of different beer addition levels on the physicochemical properties, volatile flavor compounds, and microbial communities during the fermentation of pickled peppers. A total of 37 key flavor compounds were identified, including 24 esters, 5 aldehydes, 3 alcohols, 3 terpenes, 1 acid, and 1 ketone. At the phylum level, Firmicutes, Proteobacteria, and Ascomycota were the dominant phyla. Moreover, at the genus level, *Lactiplantibacillus* and *Wickerhamomyces* were confirmed as core functional microbes involved in the formation of key flavor compounds. Under the experimental conditions of this study, the 25 %-beer added group exhibited optimal performance, improving sensory quality and showing better fermentation potential, thus providing a theoretical basis for standardized industrial production. It should be noted that this study did not conduct detailed detection of the chemical components in Snow Beer (such as sugars, hop derivatives, and volatile compounds). This limits the in depth analysis of how specific components in beer affect the fermentation of pickled peppers, and relevant analyses can be supplemented in subsequent studies. Future research should specifically explore how individual or combined components in beer affect the flavor and microbial community of pickled peppers, and employ metatranscriptomics and metaproteomics to investigate in depth the key enzymes involved in the process.

## CRediT authorship contribution statement

**Linzhu Li:** Writing – original draft, Validation, Methodology, Data curation, Conceptualization. **Wei Su:** Writing – review & editing, Funding acquisition. **Yingchun Mu:** Writing – review & editing, Supervision. **Yu Jiao:** Data curation. **Ling Chen:** Data curation. **Huayan Luo:** Investigation.

## Declaration of competing interest

The authors declare that they have no known competing financial interests or personal relationships that could have appeared to influence the work reported in this paper.

## Data Availability

Data will be made available on request.

## Appendix A Supplementary data

Ssupplementary material Image 1
