# Supplementary material for: Effects of beer addition on the fermentation quality and flavor development of pickled peppers
Source: Food Chem X. 2025 Aug 5;29:102845. doi: 10.1016/j.fochx.2025.102845 (PMC12345330; doi:10.1016/j.fochx.2025.102845)
Supplement: Supplementary file 1 — Ssupplementary material [file mmc1.docx]

**Supplementary Material**

| **Table S1. Sensory evaluation index and standard of beer picked pepper** | | |
| --- | --- | --- |
| Sensory Evaluation Indicators | Sensory Evaluation Criteria | Sensory Evaluation Scores |
| Color and Texture (20 points) | Bright red, uniformly colored with vibrant luster; chili peppers exhibit freshness | 16-20 points |
|  | Dull in color with moderate luster; chili peppers remain fresh | 11-15 points |
|  | Dark red with superficial browning; chili peppers show staleness | 6-10 points |
|  | Dark red with severe browning; chili peppers are distinctly stale | 1-5 points |
| Aroma (20 points) | Rich and pleasant aroma, free from off-odors, characterized by mellow fermented notes and distinct beer-specific fragrance | 16-20 points |
|  | Moderate aroma, no off-odors, with faint fermented or beer-derived notes | 11-15 points |
|  | Weak aroma, lacking fermented or beer-specific attributes | 6-10 points |
|  | Unpleasant aroma with offensive odors | 1-5 points |
| Acidity (15 points) | Moderate acidity with balanced taste | 12-15 points |
|  | Excessively strong or weak acidity | 8-11 points |
|  | Excessively strong or weak acidity | 4-7 points |
|  | Overly intense or extremely mild acidity | 1-3 points |
| Shape(20 points) | Minimal juice, moderate viscosity, uniform and fine texture, no stratification | 16-20 points |
|  | Little juice, slightly flowable, uniform texture, no stratification | 11-15 points |
|  | Slightly more juice, flowable, uneven texture, slight stratification | 6-10 points |
|  | Excessive juice, uneven texture, severe stratification | 1-5 points |
| Crispness (25 points) | Crisp and tender, good chewiness, no separation of flesh and skin | 20-25 points |
|  | Relatively crisp and tender, good chewiness, no separation of flesh and skin | 14-19 points |
|  | Soft and mushy, poor chewiness, but no separation of flesh and skin | 8-13 points |
|  | Soft and mushy, poor chewiness, with separation of flesh and skin | 1-7 points |

| **Table S2. Relative Contents and Types of Volatile Flavor Compounds During Fermentation of Pickled Peppers in KB Group** | | | | | | | | |
| --- | --- | --- | --- | --- | --- | --- | --- | --- |
| CAS | Compounds | relative content（μg/kg） | | | | | | |
|  |  | KB0d | KB2d | KB6d | KB11d | KB17d | KB23d | KB30d |
|  | **Esters** | **7 Kinds** | **10 Kinds** | **12 Kinds** | **14 Kinds** | **14 Kinds** | **11 Kinds** | **15 Kinds** |
| 108-05-4 | Vinyl acetate | 8.61±0.99^c^ | 278.22±10.31^a^ | 118.84±36.80^b^ | 15.72±3.37^c^ | ND | ND | ND |
|  |  |  |  |  |  |  |  |  |
| 141-78-6 | Ethyl acetate | ND | 245.61±1.10^d^ | 591.36±41.93^c^ | 408.33±26.02^c^ | 465.31±99.09^cd^ | 1145.96±194.66^b^ | 2646.22±160.55^a^ |
|  |  |  |  |  |  |  |  |  |
| 1731-84-6 | Methyl nonanoate | 92.45±1.32^a^ | ND | ND | ND | ND | ND | ND |
|  |  |  |  |  |  |  |  |  |
| 119-36-8 | Methyl salicylate | 1839.39±127.98^a^ | 555.53±90.29^b^ | 403.25±31.20^c^ | 260.18±27.79^d^ | 180.14±20.58^de^ | 110.26±11.86^e^ | 286.47±21.88^d^ |
|  |  |  |  |  |  |  |  |  |
|  |  |  |  |  |  |  |  |  |
| 10032-15-2 | Hexyl 2-methylbutyrate | 38.74±3.67^b^ | 30.94±7.22^bc^ | ND | 24.17±2.29^cd^ | 19.30±1.176^d^ | 39.06±8.04^b^ | 79.32±7.48^a^ |
|  |  |  |  |  |  |  |  |  |
| 112-39-0 | Methyl palmitate | 673.70±34.93^a^ | 197.74±51.72^c^ | 350.17±20.391^b^ | 47.07±7.63^d^ | 35.79±11.52^de^ | ND | 67.63±7.09^d^ |
|  |  |  |  |  |  |  |  |  |
| 628-97-7 | Ethyl palmitate | 38.81±7.06^e^ | 204.03±43.74^d^ | 474.71±42.28^b^ | 390.46±12.48^bc^ | 372.94±60.77^bc^ | 291.67±57.98^cd^ | 1056.61±135.69^a^ |
|  |  |  |  |  |  |  |  |  |
| 54546-22-4 | 9-Hexadecenoic acid,ethyl este | ND | 21.05±10.14^bc^ | 33.27±2.86^ab^ | 24.27±2.68^bc^ | 16.36±2.21^c^ | 43.53±11.20^a^ | 41.10±9.57^a^ |
|  |  |  |  |  |  |  |  |  |
| 111-11-5 | Methyl octanoate | ND | 81.21±19.68^a^ | ND | ND | ND | ND | ND |
|  |  |  |  |  |  |  |  |  |
| 123-29-5 | Ethyl nonanoate | ND | 133.93±31.28^c^ | 161.27±26.05^c^ | 145.03±3.16^c^ | 118.12±13.76^c^ | 212.61±45.69^b^ | 502.40±26.35^a^ |
|  |  |  |  |  |  |  |  |  |
|  |  |  |  |  |  |  |  |  |
| 76649-16-6 | Ethyl trans-4-decenoate | ND | 241.04±64.01^c^ | 335.92±35.11^c^ | ND | 248.13±8.50^c^ | 557.35±98.90^b^ | 1657.58±123.71^a^ |
|  |  |  |  |  |  |  |  |  |
| 123-92-2 | Isoamyl acetate | ND | ND | 354.88±1.90^c^ | 784.52±81.27^a^ | 560.94±39.10^b^ | 299.70±19.23^c^ | 586.38±2.46^b^ |
|  |  |  |  |  |  |  |  |  |
|  |  |  |  |  |  |  |  |  |
| 142-92-7 | Hexyl acetate | ND | ND | 307.11±20.40^d^ | 596.05±56.21^a^ | 471.14±9.16^b^ | 413.34±33.78^c^ | 487.41±7.85^b^ |
|  |  |  |  |  |  |  |  |  |
| 112-62-9 | Methyl oleate | ND | ND | ND | 106.79±7.90^a^ | ND | ND | ND |
|  |  |  |  |  |  |  |  |  |
| 628-63-7 | Amyl acetate | ND | ND | ND | 19.15±3.43^a^ | ND | ND | ND |
|  |  |  |  |  |  |  |  |  |
| 118-61-6 | Ethyl salicylate | ND | ND | ND | 60.69±17.26^b^ | 60.50±7.31^b^ | ND | 183.96±26.41^a^ |
|  |  |  |  |  |  |  |  |  |
| 106-32-1 | Ethyl caprylate | ND | ND | ND | ND | ND | 64.21±6.66^b^ | 92.57±6.79^a^ |
|  |  |  |  |  |  |  |  |  |
| 103-45-7 | Phenethyl acetate | ND | ND | 249.90±40.21^a^ | ND | ND | 132.07±42.98^b^ | 92.57±6.79^c^ |
| 124-06-1 | Ethyl myristate | ND | ND | 35.10±8.32^b^ | 26.60±1.72^b^ | 28.47±1.433^b^ | ND | 160.76±28.28^a^ |
|  |  |  |  |  |  |  |  |  |
| 544-35-4 | Ethyl linoleate | 46.20±8.78b | ND | ND | ND | ND | ND | 389.08±43.26^a^ |
|  |  |  |  |  |  |  |  |  |
| 6114-18-7 | Elaidic acid Ethyl ester | ND | ND | ND | ND | 107.79±22.29^a^ | ND | ND |
|  |  |  |  |  |  |  |  |  |
| 41114-00-5 | Pentadecanoic acid,ethyl ester | ND | ND | ND | ND | 21.32±1.07^a^ | ND | ND |
|  |  |  |  |  |  |  |  |  |
|  | **Alcohols** | **4 Kinds** | **6 Kinds** | **6 Kinds** | **4 Kinds** | **6 Kinds** | **7 Kinds** | **5 Kinds** |
| 71-41-0 | 1-Pentanol | ND | 96.60±3.62^d^ | 104.82±5.44^d^ | 120.04±18.44^cd^ | 151.04±14.97^c^ | 212.56±46.61^b^ | 262.34±0.67^a^ |
|  |  |  |  |  |  |  |  |  |
| 626-89-1 | 4-Methyl-1-pentanol | 145.57±30.73^d^ | 470.80±51.41^c^ | 565.19±25.92^b^ | 407.16±55.67^c^ | 440.24±6.84^c^ | 772.46±36.46^a^ | 623.71±6.66^b^ |
|  |  |  |  |  |  |  |  |  |
| 108-93-0 | Cyclohexanol | 188.86±68.72^a^ | ND | ND | ND | ND | ND | ND |
|  |  |  |  |  |  |  |  |  |
| 36653-82-4 | 1-Hexadecanol | 88.34±31.94^a^ | 26.39±4.64^b^ | ND | ND | 67.06±26.93^a^ | 26.43±7.379^b^ | ND |
|  |  |  |  |  |  |  |  |  |
| 696-71-9 | Cyclooctanol | ND | 216.92±53.19^a^ | 218.65±35.01^a^ | ND | ND | ND | ND |
|  |  |  |  |  |  |  |  |  |
| 629-76-5 | 1-Pentadecanol | ND | 1464.90±257.51^c^ | 1971.92±404.22^b^ | 1469.04±64.89^c^ | 1569.02±111.69^c^ | 2756.47±308.034^a^ | ND |
|  |  |  |  |  |  |  |  |  |
| 111-27-3 | Hexyl alcohol | ND | 187.77±30.20^b^ | 352.86±36.08^a^ | ND | ND | 66.24±14.75^c^ | ND |
|  |  |  |  |  |  |  |  |  |
| 78-70-6 | Linalool | ND | ND | 423.56±202.25^bc^ | 394.89±25.11^c^ | 509.58±20.80^bc^ | 573.83±92.86^b^ | 1013.48±20.45^a^ |
|  |  |  |  |  |  |  |  |  |
| 543-49-7 | 2-Heptanol | ND | ND | ND | ND | 30.71±6.03^a^ | ND | ND |
|  |  |  |  |  |  |  |  |  |
| 10482-56-1 | (-)-alpha-Terpineol | ND | ND | ND | ND | ND | 90.85±16.64^b^ | 238.48±5.53^a^ |
|  |  |  |  |  |  |  |  |  |
| 1960-12-8 | Phenylethyl alcohol | ND | ND | ND | ND | ND | ND | 1590.94±78.16^a^ |
|  |  |  |  |  |  |  |  |  |
| 112-72-1 | 1-Tetradecanol | 253.03±223.37^a^ | ND | ND | ND | ND | ND | ND |
|  |  |  |  |  |  |  |  |  |
|  | **Ketones** | **2 Kinds** | **4 Kinds** | **2 Kinds** | **3 Kinds** | **1 Kind** | **3 Kinds** | **4 Kinds** |
| 127-41-3 | α-Ionone | 54.72±5.91^a^ | ND | ND | ND | ND | ND | ND |
|  |  |  |  |  |  |  |  |  |
| 96-22-0 | 3-Pentanone | ND | 19.25±6.98^b^ | 87.89±8.34^a^ | 5.21±1.04^c^ | ND | ND | ND |
|  |  |  |  |  |  |  |  |  |
| 17283-81-7 | Dihydro-beta-ionone | ND | 22.29±7.31^c^ | ND | 20.59±4.78^c^ | ND | 70.31±16.94^b^ | 274.30±24.54^a^ |
|  |  |  |  |  |  |  |  |  |
| 23726-93-4 | Beta-damascenone | ND | ND | ND | 27.02±4.60^b^ | 36.55±8.377^b^ | 32.79±12.76^b^ | 152.54±17.24^a^ |
|  |  |  |  |  |  |  |  |  |
| 31499-72-6 | Dihydro-alpha-violetone | ND | ND | ND | ND | ND | 27.58±1.50^b^ | 106.89±7.34^a^ |
|  |  |  |  |  |  |  |  |  |
| 513-86-0 | 3-Hydroxy-2-butanone | ND | 85.53±75.003^a^ | 73.23±36.74^a^ | ND | ND | ND | 17.87±2.70^b^ |
|  |  |  |  |  |  |  |  |  |
| 1629-58-9 | 1-Penten-3-One | ND | 42.74±2.65^a^ | ND | ND | ND | ND | ND |
|  |  |  |  |  |  |  |  |  |
| 107-87-9 | 2-Pentanone | 8.81±0.60^a^ | ND | ND | ND | ND | ND | ND |
|  |  |  |  |  |  |  |  |  |
|  | **Terpenes** | **5 Kinds** | **3 Kinds** | **2 Kinds** | **5 Kinds** | **3 Kinds** | **3 Kinds** | **7 Kinds** |
| 5989-8-2 | (+)-a-Longipinene | 273.68±48.77^b^ | 209.33±51.86^bc^ | ND | 194.46±28.94^c^ | 198.27±1.21^bc^ | 255.25±47.00^bc^ | 669.54±60.49^a^ |
|  |  |  |  |  |  |  |  |  |
| 515-13-9 | β-elemene | 105.79±29.41^a^ | 63.28±33.82^b^ | ND | ND | ND | ND | ND |
|  |  |  |  |  |  |  |  |  |
| 4630-7-3 | (+)-Valencene | 11.55±3.31318^b^ | ND | ND | ND | ND | ND | 31.42±6.09743^a^ |
|  |  |  |  |  |  |  |  |  |
| 100-42-5 | Phenylethylene | ND | 37.18±11.64^b^ | ND | 42.20±0.70^b^ | ND | 19.71±18.96^c^ | 59.24±.706^a^ |
|  |  |  |  |  |  |  |  |  |
| 87-44-5 | l-Caryophyllene | ND | ND | 275.98±39.47^a^ | 246.22±20.11^b^ | ND | ND | ND |
|  |  |  |  |  |  |  |  |  |
| 5989-27-5 | D-limonene | 15.36±0.19^d^ | ND | 43.44±12.10^ab^ | 36.83±2.47^b^ | 48.58±1.305^a^ | 26.07±5.53^c^ | 36.61±1.09^b^ |
| 464-17-5 | 2-Bornene | ND | ND | ND | 14.77±0.31^c^ | 30.93±2.23^b^ | ND | 44.61±4.04^a^ |
|  |  |  |  |  |  |  |  |  |
| 99-86-5 | α-terpinene | ND | ND | ND | ND | ND | ND | 17.70±2.98^a^ |
|  |  |  |  |  |  |  |  |  |
| 489-39-4 | (+)-Aromadendrene | 722.36±73.10^b^ | ND | ND | ND | ND | ND | 2231.36±186.71^a^ |
|  |  |  |  |  |  |  |  |  |
|  | **Aldehydes** | **6 Kinds** | **5 Kinds** | **6 Kinds** | **2 Kinds** | **1 Kind** | **1 Kind** | **3 Kinds** |
| 66-25-1 | Hexanal | 82.47±17.75^b^ | 60.83±2.88^c^ | 123.88±17.21^a^ | ND | ND | ND | ND |
|  |  |  |  |  |  |  |  |  |
| 122-78-1 | Phenylacetaldehyde | 87.31±11.93^b^ | 91.31±24.25^b^ | 134.42±26.96^a^ | 14.29±0.178^cd^ | ND | 38.97±11.35^c^ | 48.03±29.54^c^ |
|  |  |  |  |  |  |  |  |  |
| 124-19-6 | Nonanal | 70.40±20.50^b^ | ND | 100.63±12.97^a^ | ND | ND | ND | ND |
|  |  |  |  |  |  |  |  |  |
| 2765-11-9 | Pentadecanal | 1481.86±95.07^a^ | 620.21±34.32^b^ | 562.38±91.01^b^ | 316.22±2.27^c^ | 235.44±11.95^c^ | ND | 1521.18±193.86^a^ |
|  |  |  |  |  |  |  |  |  |
| 629-80-1 | Hexadecanal | 67.44±1.54^a^ | ND | ND | ND | ND | ND | 67.81±10.17^a^ |
|  |  |  |  |  |  |  |  |  |
| 100-52-7 | Benzaldehyde | ND | 64.31±28.14^b^ | 114.97±52.98^a^ | ND | ND | ND | ND |
|  |  |  |  |  |  |  |  |  |
| 2548-87-0 | (E)-2-Octenal | ND | 122.79±23.09^b^ | 329.53±76.58^a^ | ND | ND | ND | ND |
|  |  |  |  |  |  |  |  |  |
| 18829-56-6 | (E)-2-Nonenal | ND | ND | ND | ND | ND | ND | ND |
|  |  |  |  |  |  |  |  |  |
| 505-57-7 | Hex-2-enal | 153.34±24.71^a^ | ND | ND | ND | ND | ND | ND |
|  |  |  |  |  |  |  |  |  |
|  | **Alkanes** | **2 Kinds** | **4 Kinds** | **3 Kinds** | **2 Kinds** | **2 Kinds** | **3 Kinds** | **3 Kinds** |
| 629-50-5 | Tridecane | 65.09±16.71^b^ | 91.06±12.63^b^ | 42.50±3.83^b^ | 24.23±1.75^b^ | 45.04±5.20^b^ | 29.76±1.94^b^ | 1218.10±106.80^a^ |
|  |  |  |  |  |  |  |  |  |
| 2882-96-4 | 3-Methylpentadecane | 268.61±48.17^b^ | 122.24±37.32^cd^ | 139.56±28.92^cd^ | 96.55±6.15^d^ | 98.63±5.75^d^ | 176.5480±42.03547^c^ | 629.71±46.69^a^ |
|  |  |  |  |  |  |  |  |  |
| 629-92-5 | Nonadecane | ND | 11.95±3.16^d^ | 14.96±0.73^c^ | ND | ND | 20.13±2.06^b^ | 47.68±0.34^a^ |
|  |  |  |  |  |  |  |  |  |
| 1120-21-4 | Undecane | ND | 196.40±22.49^a^ | ND | ND | ND | ND | ND |
|  |  |  |  |  |  |  |  |  |
|  | **Phenolics** | **0** | **0** | **0** | **1 Kind** | **1 Kind** | **0** | **0** |
| 1195-09-1 | 2-Methoxy-5-methylphenol | ND | ND | ND | 311.05±29.93^b^ | 458.04±24.65^a^ | ND | ND |
|  |  |  |  |  |  |  |  |  |
|  | **Acid** | **0** | **1 Kind** | **1 Kind** | **0** | **1 Kind** | **0** | **0** |
| 64-19-7 | Acetic acid | ND | 1395.49±185.10^b^ | 2602.13±316.67^a^ | ND | 958.96±101.94^c^ | ND | ND |
|  |  |  |  |  |  |  |  |  |
|  | **Others** | **1 Kind** | **0 Kind** | **2 Kinds** | **2 Kinds** | **2 Kinds** | **1 Kind** | **1 Kind** |
| 3777-69-3 | 2-Pentylfuran | 44.35±4.18^c^ | ND | 410.46±34.58^a^ | 120.48±15.98^b^ | 127.79±4.92^b^ | ND | ND |
|  |  |  |  |  |  |  |  |  |
| 24683-00-9 | 2-Methoxy-3-isobutyl pyrazine | ND | ND | 92.62±7.03^bc^ | 80.66±1.924^c^ | 66.22±5.25^d^ | 102.73±15.45^b^ | 209.43±5.84^a^ |
|  |  |  |  |  |  |  |  |  |

Note: Different letters in the same row indicate significant differences (*p* < 0.05, Confidence Interval ：CI_95%_) and “ ND” indicates that the substance was not detected.

**Table S3. Relative Contents and Types of Volatile Flavor Compounds During Fermentation of Pickled Peppers in 25% beer-added Group**

| CAS | Compounds | Relative content（μg/kg） | | | | | | |
| --- | --- | --- | --- | --- | --- | --- | --- | --- |
|  |  | 25%0d | 25%2d | 25%6d | 25%11d | 25%17d | 25%23d | 25%30d |
|  | **Esters** | **7 Kinds** | **18 Kinds** | **19 Kinds** | **16 Kinds** | **18 Kinds** | **20 Kinds** | **18 Kinds** |
| 108-05-4 | Vinyl acetate | ND | 61.86±3.01^a^ | 34.07±10.78^b^ | ND | ND | ND | ND |
|  |  |  |  |  |  |  |  |  |
| 141-78-6 | Ethyl acetate | 41.36±12.61^e^ | 968.69±17.814^c^ | 493.81±51.29^d^ | 1244.47±259.50^c^ | 1671.78±170.964^b^ | 1807.35±257.83^b^ | 3055.43±491.97^a^ |
|  |  |  |  |  |  |  |  |  |
| 119-36-8 | Methyl salicylate | 1924.00±80.11^b^ | 2216.86±261.36^a^ | 1406.13±100.47^c^ | 1158.26±76.99^d^ | 481.45±111.56^e^ | 483.87±73.12^e^ | 572.41±47.97^e^ |
|  |  |  |  |  |  |  |  |  |
|  |  |  |  |  |  |  |  |  |
| 112-39-0 | Methyl palmitate | 776.91±194.22^b^ | 735.37±99.08^b^ | 1336.63±153.74^a^ | 835.61±89.59^b^ | 1533.92±503.03^a^ | 832.05±62.31^b^ | 164.38±53.95^c^ |
|  |  |  |  |  |  |  |  |  |
| 628-97-7 | Ethyl palmitate | 263.77±91.11^e^ | 2172.64±215.11d^e^ | 3080.75±302.61^d^ | 5864.17±668.28^bc^ | 4402.35±1573.67^cd^ | 11770.16±3072.81^a^ | 7599.89±980.74^b^ |
|  |  |  |  |  |  |  |  |  |
| 54546-22-4 | 9-Hexadecenoic acid,ethyl este | ND | 264.96±22.38^d^ | 373.52±43.99^c^ | 437.18±57.64^c^ | 108.61±49.90^e^ | 1081.50±35.43^a^ | 559.72±121.70^b^ |
|  |  |  |  |  |  |  |  |  |
| 111-11-5 | Methyl octanoate | ND | 186.79±27.44^a^ | 197.59±27.00^a^ | ND | ND | ND | ND |
|  |  |  |  |  |  |  |  |  |
| 123-29-5 | Ethyl nonanoate | ND | 966.14±57.58^bc^ | 1040.67±93.92^b^ | 1048.92±238.90^b^ | 735.47±78.35^c^ | 1306.25±204.64^a^ | 971.54±101.12^bc^ |
|  |  |  |  |  |  |  |  |  |
|  |  |  |  |  |  |  |  |  |
| 76649-16-6 | Ethyl-trans-4-Decenoate | ND | 2349.33±222.46^b^ | 2837.60±301.29^b^ | 2683.57±606.61^b^ | 2497.87±146.80^b^ | 3769.44±142.57^a^ | 3060.16±823.00^ab^ |
|  |  |  |  |  |  |  |  |  |
| 123-92-2 | Isoamyl acetate | 73.36±19.66^b^ | 96.19±7.31^ab^ | ND | ND | ND | 111.65±34.51^ab^ | 136.51±23.68^a^ |
|  |  |  |  |  |  |  |  |  |
| 118-61-6 | Ethyl salicylate | ND | 497.72±18.50^b^ | 481.79±37.59^b^ | 737.77±87.21^a^ | 502.64±128.73^b^ | 838.99±26.71^a^ | 575.87±80.17^b^ |
|  |  |  |  |  |  |  |  |  |
| 106-32-1 | Ethyl caprylate | ND | 597.60±10.28^c^ | 573.02±54.42^cd^ | 494.32±84.41^de^ | 449.13±73.48^e^ | 1688.49±24.64^a^ | 668.66±36.87^b^ |
|  |  |  |  |  |  |  |  |  |
| 103-45-7 | Phenethyl acetate | ND | ND | ND | ND | ND | ND | 190.70±76.00^a^ |
|  |  |  |  |  |  |  |  |  |
| 124-06-1 | Ethyl myristate | ND | 244.95±23.29^de^ | 306.12±19.80^cd^ | ND | 550.95±140.89^bc^ | 1212.49±341.67^a^ | 681.79±114.38^b^ |
|  |  |  |  |  |  |  |  |  |
| 544-35-4 | Ethyl linoleate | 240.92±69.41^d^ | 1451.80±164.23^b^ | 1862.27±195.20^c^ | 3478.96±404.60^b^ | 3526.79±878.49^b^ | 7470.77±115.32^a^ | 3376.02±359.26^b^ |
|  |  |  |  |  |  |  |  |  |
| 110-38-3 | Ethyl caprate | ND | 135.48±13.26^c^ | 167.11±67.00^bc^ | 132.10±22.34^c^ | 166.57±27.88^bc^ | 489.76±46.39^a^ | 229.87±27.60^b^ |
|  |  |  |  |  |  |  |  |  |
| 6114-18-7 | Eladic acid ethyl ester | ND | 1019.70±105.32^d^ | 1380.87±171.83^cd^ | 2229.12±227.18^bc^ | 2614.47±787.99^b^ | 4470.74±1037.70^a^ | 2367.84±291.56^b^ |
|  |  |  |  |  |  |  |  |  |
| 93-89-0 | Ethyl benzoate | ND | ND | 88.63±8.52^b^ | 91.52±34.90^b^ | ND | 132.97±13.45^a^ | ND |
|  |  |  |  |  |  |  |  |  |
| 112-63-0 | Methyl linoleate | ND | 197.67±34.31^d^ | 417.21±46.81^b^ | 276.14±35.56^c^ | 1174.87±36.72^a^ | 406.77±65.02^b^ | ND |
|  |  |  |  |  |  |  |  |  |
| 111-61-5 | Ethyl stearate | ND | ND | 88.99±19.52^c^ | 150.22±6.23^c^ | 129.29±25.10^bc^ | 305.24±94.00^a^ | 171.30±21.24^b^ |
|  |  |  |  |  |  |  |  |  |
| 106-33-2 | Ethyl laurate | ND | ND | ND | 313.67±65.52^b^ | 209.59±61.07^b^ | 583.04±142.47^a^ | 289.47±8.01^b^ |
|  |  |  |  |  |  |  |  |  |
| 627-90-7 | Ethyl undecanoate | ND | ND | 207.60±27.23^b^ | ND | 106.35±16.02^c^ | 313.41±63.63^a^ | 208.97±34.59^b^ |
|  |  |  |  |  |  |  |  |  |
| 41114-00-5 | Pentadecanoic acid,ethyl ester | 38.68±8.58^b^ | ND | ND | ND | ND | 83.01±25.50^a^ | ND |
|  |  |  |  |  |  |  |  |  |
| 110-42-9 | Methyl decanoate | ND | 127.43±10.09^a^ | ND | ND | ND | ND | ND |
|  |  |  |  |  |  |  |  |  |
|  | **Alcohols** | **3 Kinds** | **6 Kinds** | **5 Kinds** | **6 Kinds** | **8 Kinds** | **5 Kinds** | **6 Kinds** |
| 71-41-0 | 1-Pentanol | ND | ND | 527.88±119.91^c^ | ND | ND | 1404.02±164.73^a^ | 733.66±24.04^b^ |
|  |  |  |  |  |  |  |  |  |
| 626-89-1 | 4-Methyl-1-pentanol | 297.53±56.14^b^ | 403.91±28.47^a^ | 423.29±44.58^a^ | 487.80±44.47^a^ | 400.16±52.98^a^ | 450.04±46.82^a^ | 468.00±44.29^a^ |
|  |  |  |  |  |  |  |  |  |
| 696-71-9 | Cyclooctanol | ND | 293.99±68.79168^c^ | 390.52±89.63^b^ | 506.69±7.21^a^ | 308.23±46.73^c^ | ND | ND |
|  |  |  |  |  |  |  |  |  |
| 629-76-5 | 1-Pentadecanol | 5121.25±95.62^a^ | ND | ND | ND | ND | 5440.17±234.49^a^ | 4298.88±856.82^b^ |
|  |  |  |  |  |  |  |  |  |
| 111-27-3 | Hexyl alcohol | ND | 112.60±35.48b^c^ | 204.34±21.05^b^ | 190.68±23.78^b^ | 387.64±168.27^a^ | ND | ND |
|  |  |  |  |  |  |  |  |  |
| 78-70-6 | Linalool | 151.53±22.18^d^ | 201.67±20.06^d^ | ND | 426.53±33.51^c^ | 521.79±85.73^b^ | 568.79±11.82^b^ | 745.45±27.13^a^ |
|  |  |  |  |  |  |  |  |  |
| 10482-56-1 | α-terpineol | ND | ND | ND | ND | 117.03±17.73b | ND | 205.20±18.92a |
|  |  |  |  |  |  |  |  |  |
| 123-51-3 | Isoamyl alcohol | ND | 480.43±70.06^b^ | ND | 676.58±75.85^a^ | 304.84±227.78^c^ | ND | ND |
|  |  |  |  |  |  |  |  |  |
| 1960-12-8 | Phenylethyl alcohol | ND | 2544.61±59.73^d^ | 2234.64±185.48^d^ | 3380.53±521.59^c^ | 3604.96±439.57^bc^ | 6868.09±987.30^a^ | 4332.96±259.24^b^ |
|  |  |  |  |  |  |  |  |  |
| 40716-66-3 | Nerolidol | ND | ND | ND | ND | 1073.23±172.62^a^ | ND | ND |
|  |  |  |  |  |  |  |  |  |
|  | **Ketones** | **1 Kind** | **1 Kind** | **1 Kind** | **2 Kinds** | **1 Kind** | **1 Kind** | **1 Kind** |
| 127-41-3 | α-ionone | 86.27±20.29^a^ | ND | ND | ND | ND | ND | ND |
|  |  |  |  |  |  |  |  |  |
| 23726-93-4 | Beta-damascenone | ND | ND | ND | ND | 59.11±9.37^b^ | 70.15±15.42^b^ | 151.53±0.36^a^ |
|  |  |  |  |  |  |  |  |  |
| 513-86-0 | 3-Hydroxy-2-butanone | ND | 165.25±8.51^a^ | 73.00±1.92^b^ | 74.29±16.35^b^ | ND | ND | ND |
|  |  |  |  |  |  |  |  |  |
| 1629-58-9 | 1-Penten-3-one | ND | ND | ND | 199.71±45.00^a^ | ND | ND | ND |
|  |  |  |  |  |  |  |  |  |
|  | **Terpenes** | **3 Kinds** | **1 Kind** | **3 Kinds** | **2 Kinds** | **4 Kinds** | **3 Kinds** | **5 Kinds** |
| 5989-8-2 | α-Longipinene | 394.02±85.70^c^ | 607.64±22.16^a^ | 508.98±28.517^b^ | ND | 375.77±45.64^c^ | 508.52±65.52^b^ | 611.01±13.32^a^ |
|  |  |  |  |  |  |  |  |  |
| 515-13-9 | β-elemene | 56.42±1.03^c^ | ND | 96.65±19.70^b^ | 126.43±33.42^b^ | ND | 179.73±29.08^a^ | 115.7667±18.87^b^ |
|  |  |  |  |  |  |  |  |  |
| 100-42-5 | Phenylethylene | ND | ND | ND | 83.16±36.12^b^ | 42.41±12.58^bc^ | 498.64±57.34^a^ | 90.12±0.85^b^ |
|  |  |  |  |  |  |  |  |  |
| 87-44-5 | l-Caryophyllene | 733.07±88.74^b^ | ND | 1377.53±103.43^a^ | ND | ND | ND | ND |
|  |  |  |  |  |  |  |  |  |
| 5989-27-5 | D-limonene | ND | ND | ND | ND | 21.74±1.312^b^ | ND | 31.82±8.65^a^ |
| 99-86-5 | α-terpinene | ND | ND | ND | ND | ND | ND | 54.8393±12.75^a^ |
|  |  |  |  |  |  |  |  |  |
| 489-39-4 | (+)-Aromadendrene | ND | ND | ND | ND | 1113.30±29.16^a^ | ND | ND |
|  |  |  |  |  |  |  |  |  |
|  | **Aldehydes** | **7 Kinds** | **6 Kinds** | **6 Kinds** | **7 Kinds** | **4 Kinds** | **5 Kinds** | **5 Kinds** |
| 66-25-1 | Hexanal | 60.05±5.84^c^ | 209.89±25.82^b^ | 182.99±32.99^b^ | 598.48±74.62^a^ | ND | 243.22±20.50^b^ | 54.71±10.07^c^ |
|  |  |  |  |  |  |  |  |  |
| 122-78-1 | Phenylacetaldehyde | 196.70±56.00^bc^ | 179.97±77.25b^c^ | 154.08±63.64b^c^ | 420.71±149.03^a^ | 250.00±41.93^b^ | 217.81±38.05^bc^ | 101.19±29.67^c^ |
|  |  |  |  |  |  |  |  |  |
| 124-19-6 | Nonanal | 47.40±8.07^d^ | ND | ND | 123.21±20.22^c^ | 145.39±18.91^b^ | 174.71±1.17^a^ | ND |
|  |  |  |  |  |  |  |  |  |
| 2765-11-9 | Pentadecanal | 2662.43±49.62^ab^ | 3105.96±172.74^a^ | 2306.12±150.36^b^ | 2108.21±353.00^b^ | 1467.68±295.64^c^ | 2179.71±661.79^b^ | 1355.60±45.88^c^ |
|  |  |  |  |  |  |  |  |  |
| 629-80-1 | Hexadecanal | 127.70±19.07^ab^ | 136.47±5.41^a^ | 106.60±12.86^cd^ | 93.76±11.65^d^ | ND | 114.88±2.78^bc^ | 58.91±6.46^e^ |
|  |  |  |  |  |  |  |  |  |
| 100-52-7 | Benzaldehyde | ND | ND | 74.11±15.94^c^ | 190.06±37.07^b^ | 228.44±23.96^a^ | ND | ND |
|  |  |  |  |  |  |  |  |  |
| 2548-87-0 | (E)-2-Octenal | 118.01±55.40^c^ | 374.49±22.99^b^ | 381.26±21.14^b^ | 1264.40±134.66^a^ | ND | ND | 298.02±93.00^b^ |
|  |  |  |  |  |  |  |  |  |
| 18829-56-6 | (E)-2-Nonenal | 561.89±272.17^a^ | 194.38±41.38^b^ | ND | ND | ND | ND | ND |
|  |  |  |  |  |  |  |  |  |
|  | **Alkanes** | **4 Kinds** | **2 Kinds** | **2 Kinds** | **2 Kinds** | **2 Kinds** | **2 Kinds** | **3 Kinds** |
| 629-50-5 | Tridecane | 53.40±11.16^d^ | ND | ND | 70.45±2.82^c^ | 317.64±10.08^a^ | ND | 111.38±12.57^b^ |
|  |  |  |  |  |  |  |  |  |
| 2882-96-4 | 3-Methylpentadecane | 302.55±54.61^c^ | 565.14±50.81^a^ | 523.26±8.68^a^ | ND | 331.85±62.812^bc^ | 536.65±27.42^a^ | 391.02±66.83^b^ |
|  |  |  |  |  |  |  |  |  |
| 629-92-5 | Nonadecane | 50.68±11.28^cd^ | 74.59±6.23^ab^ | 58.34±5.377^c^ | 47.55±19.81^cd^ | ND | 76.91±1.48^a^ | 37.71±5.19^d^ |
|  |  |  |  |  |  |  |  |  |
| 1120-21-4 | Undecane | 108.17±17.40^a^ | ND | ND | ND | ND | ND | ND |
|  |  |  |  |  |  |  |  |  |
|  | **Phenolics** | **0** | **0** | **0** | **0** | **0** | **2 Kinds** | **0** |
| 1195-09-1 | 2-Methoxy-5-Methylphenol | ND | ND | ND | ND | ND | 1293.96±339.60^a^ | ND |
|  |  |  |  |  |  |  |  |  |
| 2785-89-9 | 4-Ethyl-2-methoxyphenol | ND | ND | ND | ND | ND | 479.02±9.58^a^ | ND |
|  |  |  |  |  |  |  |  |  |
|  | **Acid** | **0** | **0** | **1 Kind** | **0** | **0** | **0** | **0** |
| 64-19-7 | Acetic acid | ND | ND | 167.47±71.67^a^ | ND | ND | ND | ND |
|  |  |  |  |  |  |  |  |  |
|  | **Others** | **1 Kind** | **2 Kinds** | **2 Kinds** | **2 Kinds** | **2 Kinds** | **1 Kind** | **2 Kinds** |
| 3777-69-3 | 2-Pentylfuran | ND | 395.14±49.09^a^ | 397.78±16.52^a^ | 378.59±28.62^a^ | 88.41±17.90^c^ | 237.99±19.95^b^ | 81.30±24.18^c^ |
|  |  |  |  |  |  |  |  |  |
| 24683-00-9 | 2-Methoxy-3-isobutyl pyrazine | 198.23±67.78^abc^ | 247.60±12.71^a^ | 186.62±22.33^bc^ | 236.01±4.38^ab^ | 110.93±15.05^d^ | ND | 148.26±16.51^cd^ |
|  |  |  |  |  |  |  |  |  |

Note: Different letters in the same row indicate significant differences (*p* < 0.05, Confidence Interval ：CI_95%_) and “ ND” indicates that the substance was not detected.

**Table S4. Relative Contents and Types of Volatile Flavor Compounds During Fermentation of Pickled Peppers in 50% beer-added Group**

| CAS | compounds | relative content（μg/kg） | | | | | | |
| --- | --- | --- | --- | --- | --- | --- | --- | --- |
|  |  | 50%0d | 50%2d | 50%6d | 50%11d | 50%17d | 50%23d | 50%30d |
|  | **Esters** | **13 Kinds** | **22 Kinds** | **24 Kinds** | **23 Kinds** | **21 Kinds** | **20 Kinds** | **20 Kinds** |
| 108-05-4 | vinyl acetate | ND | 49.27±14.59^c^ | 205.55±18.72^a^ | 82.84±17.98^b^ | ND | 6.90±1.34^d^ | ND |
|  |  |  |  |  |  |  |  |  |
| 141-78-6 | Ethyl acetate | 69.29±9.45^e^ | 742.72±22.39^d^ | 3284.21±81.40^a^ | 2693.59±363.27^b^ | 3510.88±390.48^a^ | 1472.94±341.92^c^ | 2370.51±185.40^b^ |
|  |  |  |  |  |  |  |  |  |
| 119-36-8 | Methyl salicylate | 2415.11±516.01^a^ | 2001.62±179.39^b^ | 1076.85±126.42^c^ | 738.22±121.06^cd^ | 484.68±35.84^de^ | 310.20±62.81^e^ | 365.04±103.63^de^ |
|  |  |  |  |  |  |  |  |  |
|  |  |  |  |  |  |  |  |  |
| 112-39-0 | Methyl palmitate | 1102.26±214.84^bc^ | 2703.33±356.26^a^ | 3112.94±209.99^a^ | 1535.89±656.83^b^ | 1004.62±96.52^bc^ | 771.62±103.02^cd^ | 387.13±4.88^d^ |
|  |  |  |  |  |  |  |  |  |
| 628-97-7 | Ethyl palmitate | 402.64±40.24^d^ | 3534.69±412.10^c^ | 46.05±1.86^d^ | 4164.76±730.27^bc^ | 10776.34±1326.70^a^ | 3986.98±826.75^bc^ | 4911.46±509.21^b^ |
|  |  |  |  |  |  |  |  |  |
| 54546-22-4 | 9-Hexadecenoic acid,ethyl este | 136.16±6.66^cd^ | 92.14±9.29^d^ | 258.45±20.69^c^ | 237.52±41.03^c^ | 805.70±174.32^a^ | 98.16±16.61^d^ | 634.82±73.42^b^ |
|  |  |  |  |  |  |  |  |  |
| 111-11-5 | Methyl octanoate | ND | 415.23±95.33^a^ | 403.20±121.61^a^ | 156.95±1.02^b^ | ND | ND | ND |
|  |  |  |  |  |  |  |  |  |
| 123-29-5 | Ethyl nonanoate | ND | 1008.42±58.42^b^ | 969.93±163.38^b^ | 1040.43±73.15^b^ | 1369.37±218.79^a^ | 956.58±213.66^b^ | 1515.02±191.87^a^ |
|  |  |  |  |  |  |  |  |  |
|  |  |  |  |  |  |  |  |  |
| 76649-16-6 | Ethyl -Trans-4-decenoate | 204.05±19.11^c^ | 2599.58±106.39^b^ | 3088.24±442.12^b^ | ND | 3748.83±621.37^a^ | ND | ND |
|  |  |  |  |  |  |  |  |  |
| 123-92-2 | Isoamyl acetate | 122.39±31.13^b^ | 179.87±52.28^a^ | 87.96±15.44^bc^ | 67.28±11.45^c^ | 117.84±24.90^bc^ | ND | 125.02±25.71^b^ |
| 112-62-9 | Methyl oleate | ND | ND | ND | ND | 402.38±118.04^a^ | ND | ND |
|  |  |  |  |  |  |  |  |  |
| 118-61-6 | Ethyl salicylate | ND | 631.68±361.01^ab^ | 515.69±103.66^b^ | 604.26±79.38^b^ | 925.91±162.56^a^ | 602.58±96.75^b^ | 669.66±38.48^ab^ |
|  |  |  |  |  |  |  |  |  |
| 106-32-1 | Ethyl caprylate | 113.75±21.52^e^ | 1227.68±74.37^cd^ | 2484.93±356.44^a^ | 1646.13±174.88^b^ | 1232.66±240.74^cd^ | 1098.94±302.38^d^ | 1622.41±159.77^bc^ |
|  |  |  |  |  |  |  |  |  |
| 103-45-7 | Phenethyl acetate | ND | 218.92±18.281^ab^ | 223.53±1.27^ab^ | 268.22±39.28^a^ | ND | 131.85±32.59^c^ | 197.91±79.08^b^ |
|  |  |  |  |  |  |  |  |  |
| 124-06-1 | Ethyl myristate | ND | 464.66±98.16^c^ | 705.71±147.65^b^ | 669.36±119.74^b^ | 1133.95±111.06^a^ | 587.81±87.71^bc^ | 717.82±70.17^b^ |
|  |  |  |  |  |  |  |  |  |
| 544-35-4 | Ethyl linoleate | 233.32±38.52^d^ | 1989.20±382.148^c^ | 5555.05±262.38^a^ | 4815.74±844.33^a^ | 5656.16±714.13^a^ | 2420.42±547.24^bc^ | 3201.27±533.49^b^ |
|  |  |  |  |  |  |  |  |  |
| 110-38-3 | Ethyl caprate | ND | 373.16±8.21^bc^ | 311.52±33.47^c^ | 419.58±37.54^b^ | 620.17±109.08^a^ | 350.58±78.84^bc^ | 424.51±31.79^b^ |
|  |  |  |  |  |  |  |  |  |
| 6114-18-7 | Elaidic acid ethyl ester | ND | 1556.10±229.96^c^ | 4945.97±277.92^a^ | 4230.24±753.13^ab^ | 3907.71±556.65^b^ | 1879.51±435.95^c^ | 2277.38±150.63^c^ |
|  |  |  |  |  |  |  |  |  |
| 93-89-0 | Ethyl benzoate | 22.62±9.40^d^ | 120.53±10.82^c^ | ND | 83.05±13.88^c^ | 130.91±42.82^bc^ | 181.64±36.90^ab^ | 207.84±64.50^a^ |
|  |  |  |  |  |  |  |  |  |
| 112-63-0 | Methyl linoleate | 379.57±101.66^cd^ | 635.77±136.15^b^ | 1190.83±197.79^a^ | 551.39±193.51^bc^ | 445.19±117.73^bc^ | 338.43±60.02^cd^ | 143.41±9.60^d^ |
|  |  |  |  |  |  |  |  |  |
| 111-61-5 | Ethyl stearate | ND | ND | 100.07±27.71^c^ | 104.08±24.66^c^ | 264.40±50.47^a^ | 184.41±72.67^b^ | 162.54±58.40^bc^ |
|  |  |  |  |  |  |  |  |  |
| 106-33-2 | Ethyl laurate | ND | 337.17±33.46^a^ | 369.98±83.031^a^ | 353.01±85.37^a^ | 516.10±102.20^a^ | 337.98±115.61^a^ | 422.13±145.22^a^ |
|  |  |  |  |  |  |  |  |  |
| 627-90-7 | Ethyl undecanoate | ND | ND | 149.98±28.863^b^ | 151.32±12.62^b^ | 258.55±56.39^a^ | 148.98±36.84^b^ | 203.87±21.95^b^ |
|  |  |  |  |  |  |  |  |  |
| 41114-00-5 | Pentadecanoic acid, ethyl ester | 65.20±4.87^a^ | ND | ND | ND | ND | 31.00±12.144^c^ | 47.17±18.60^b^ |
|  |  |  |  |  |  |  |  |  |
| 110-42-9 | Methyl decanoate | 86.42±5.74^d^ | 274.45±36.78^a^ | 252.19±31.021^ab^ | 176.35±20.28^c^ | 229.50±23.94^b^ | ND | ND |
|  |  |  |  |  |  |  |  |  |
| 124-10-7 | Methyl tetradecanoate | ND | 250.44±36.416^a^ | 203.08±9.29^b^ | 127.20±48.83^c^ | ND | ND | ND |
|  |  |  |  |  |  |  |  |  |
| 112-61-8 | Methyl stearate | ND | ND | 48.40±6.08^a^ | ND | ND | ND | ND |
|  |  |  |  |  |  |  |  |  |
|  | **Alcohols** | **5 Kinds** | **7 Kinds** | **6 Kinds** | **6 Kinds** | **7 Kinds** | **5 Kinds** | **7 Kinds** |
| 71-41-0 | 1-Pentanol | ND | 919.66±59.21^c^ | 967.51±80.16^bc^ | 866.69±12.95^c^ | 1193.57±151.40^ab^ | 1115.60±293.21^abc^ | 1324.01±100.31^a^ |
|  |  |  |  |  |  |  |  |  |
| 626-89-1 | 4-Methyl-1-pentanol | 281.83±100.31^c^ | 407.95±43.97^ab^ | 421.78±36.42^ab^ | 334.17±38.49^bc^ | 467.98±76.81^a^ | 416.53±14.58^ab^ | 442.89±16.70^a^ |
|  |  |  |  |  |  |  |  |  |
| 36653-82-4 | 1-Hexadecanol | ND | ND | 170.40±57.38^a^ | ND | ND | ND | ND |
|  |  |  |  |  |  |  |  |  |
| 696-71-9 | Cyclooctanol | ND | 146.08±26.27^a^ | 163.30±0.64^a^ | 76.92±1.90^b^ | ND | ND | ND |
|  |  |  |  |  |  |  |  |  |
| 629-76-5 | 1-pentadecanol | 3722.80±14.57^b^ | 5763.04±244.95^a^ | ND | ND | 5719.37±23.22^a^ | ND | 3766.49±128.94^b^ |
|  |  |  |  |  |  |  |  |  |
| 111-27-3 | Hexyl alcohol | ND | ND | 123.37±15.934^c^ | ND | 261.76±31.07^a^ | 148.17±45.25^bc^ | 169.69±27.70^b^ |
|  |  |  |  |  |  |  |  |  |
| 78-70-6 | Linalool | 231.98±38.70^c^ | 187.80±48.07^c^ | ND | ND | 559.56±60.40^a^ | 341.98±91.73^b^ | 523.09±70.19^a^ |
|  |  |  |  |  |  |  |  |  |
| 10482-56-1 | α-Terpineol | ND | ND | ND | ND | 75.47±3.73^a^ | ND | ND |
|  |  |  |  |  |  |  |  |  |
| 123-51-3 | Isoamyl alcohol | 174.26±5.88^b^ | 660.54±460.80^a^ | ND | ND | ND | ND | 79.57±15.82^b^ |
|  |  |  |  |  |  |  |  |  |
| 1960-12-8 | Phenylethyl alcohol | ND | 5587.75±886.03^b^ | 5537.76±403.76^b^ | 6115.70±968.78^b^ | 7412.37±365.02^a^ | 6583.29±511.60^ab^ | 6283.53±303.17^b^ |
|  |  |  |  |  |  |  |  |  |
| 98-85-1 | DL-1-Phenethylalcohol | 377.61±73.86^a^ | ND | ND | ND | ND | ND | ND |
|  |  |  |  |  |  |  |  |  |
| 40716-66-3 | Nerolidol | ND | ND | ND | 1046.34±146.72^a^ | ND | ND | ND |
|  |  |  |  |  |  |  |  |  |
| 112-72-1 | 1-Tetradecanol | ND | ND | ND | 52.90±2.27^a^ | ND | ND | ND |
|  |  |  |  |  |  |  |  |  |
|  | Ketones | 0 | 1 | 1 | 2 | 2 | 1 | 2 |
| 96-22-0 | 3-Pentanone | ND | ND | ND | 31.04±1.03^b^ | 55.95±8.41^a^ | ND | 31.90±8.53^b^ |
|  |  |  |  |  |  |  |  |  |
| 23726-93-4 | Beta-damascenone | ND | ND | ND | ND | ND | 34.16±7.08^b^ | 74.46±14.34^a^ |
|  |  |  |  |  |  |  |  |  |
| 513-86-0 | 3-Hydroxy-2-Butanone | ND | 91.10±70.94^a^ | 83.03±36.71^a^ | 51.09±11.80^ab^ | 49.23±1.29^ab^ | ND | ND |
|  |  |  |  |  |  |  |  |  |
|  | **Terpenes** | **2 Kinds** | **3 Kinds** | **3 Kinds** | **2 Kinds** | **4 Kinds** | **2 Kinds** | **5 Kinds** |
| 5989-8-2 | (+)-a-Longipinene | 449.24±35.75^d^ | 626.30±15.78^b^ | 466.80±82.01^cd^ | 401.07±42.45^d^ | 536.33±62.62^c^ | ND | 730.79±39.72^a^ |
|  |  |  |  |  |  |  |  |  |
| 515-13-9 | β-elemene | 485.24±171.23^a^ | 167.30±22.19^cde^ | 254.93±38.35^bcd^ | ND | 362.61±110.32^ab^ | 124.94±9.91^de^ | 342.33±161.84^abc^ |
|  |  |  |  |  |  |  |  |  |
| 4630-7-3 | (+)-Valencene | ND | ND | ND | ND | 184.83±38.45^a^ | ND | 119.01±49.19^b^ |
|  |  |  |  |  |  |  |  |  |
| 100-42-5 | Phenylethylene | ND | ND | 63.86±9.29^c^ | 104.96±8.44^c^ | 284.48±38.27^b^ | 259.53±61.19^b^ | 392.90±52.97^a^ |
|  |  |  |  |  |  |  |  |  |
| 87-44-5 | l-Caryophyllene | ND | 1697.93±180.64^a^ | ND | ND | ND | ND | ND |
|  |  |  |  |  |  |  |  |  |
| 464-17-5 | 2-Bornene | ND | ND | ND | ND | ND | ND | 31.60±9.57^a^ |
|  |  |  |  |  |  |  |  |  |
|  | **Aldehydes** | **7 Kinds** | **5 Kinds** | **7 Kinds** | **6 Kinds** | **6 Kinds** | **5 Kinds** | **6 Kinds** |
| 66-25-1 | Hexanal | 19.68±9.54^c^ | 51.34±6.54^c^ | 196.24±58.70^a^ | 157.69±23.22^ab^ | 209.43±109.88^a^ | 82.75±1.99^bc^ | 86.29±40.97^bc^ |
|  |  |  |  |  |  |  |  |  |
| 122-78-1 | Phenylacetaldehyde | 241.30±78.66^a^ | 189.42±74.61^a^ | 199.82±15.69^a^ | 275.92±66.238^a^ | 273.36±124.98^a^ | 155.70±23.19^a^ | 173.37±34.19^a^ |
|  |  |  |  |  |  |  |  |  |
| 124-19-6 | Nonanal | ND | ND | 138.61±17.20^b^ | 189.26±19.52^a^ | 165.68±57.05^ab^ | 128.84±30.11^b^ | 151.21±4.52^ab^ |
|  |  |  |  |  |  |  |  |  |
| 2765-11-9 | Pentadecanal | 2894.42±302.27^a^ | 2755.91±477.28^ab^ | 1410.38±128.43^c^ | 651.73±100.27^d^ | 2430.79±186.12^b^ | ND | 825.38±53.96^d^ |
|  |  |  |  |  |  |  |  |  |
| 629-80-1 | Hexadecanal | 124.63±13.43^a^ | 125.93±23.644^a^ | 79.51±33.36^b^ | ND | 103.10±26.61^ab^ | ND | ND |
|  |  |  |  |  |  |  |  |  |
| 100-52-7 | Benzaldehyde | ND | ND | ND | 109.01±26.08^a^ | ND | 116.26±34.94^a^ | 120.17±38.05^a^ |
|  |  |  |  |  |  |  |  |  |
| 2548-87-0 | (E)-2-Octenal | 131.23±53.52^c^ | 161.80±17.17^c^ | 293.57±50.73^bc^ | 420.98±41.82^ab^ | 627.59±320.02^a^ | 310.08±67.54^bc^ | 291.47±61.99^bc^ |
|  |  |  |  |  |  |  |  |  |
| 18829-56-6 | (E)-2-Nonenal | 494.80±120.03^a^ | ND | 166.1606±32.12^b^ | ND | ND | ND | ND |
|  |  |  |  |  |  |  |  |  |
| 505-57-7 | Hex-2-enal | 142.70±20.33^a^ | ND | ND | ND | ND | ND | ND |
|  |  |  |  |  |  |  |  |  |
|  | **Alkanes** | **2 Kinds** | **2 Kinds** | **2 Kinds** | **3 Kinds** | **3 Kinds** | **2 Kinds** | **2 Kinds** |
| 629-50-5 | Tridecane | 68.77±15.24^de^ | ND | 275.13±40.08^b^ | 693.38±81.93^a^ | 142.95±30.16^cd^ | 236.09±85.57^b^ | 208.36±24.57^bc^ |
|  |  |  |  |  |  |  |  |  |
| 2882-96-4 | 3-Methylpentadecane | ND | 611.58±60.28^a^ | 379.45±171.85^bc^ | 289.21±39.41^c^ | 502.11±57.52^ab^ | 280.75±82.90^c^ | 437.98±149.24^abc^ |
|  |  |  |  |  |  |  |  |  |
| 629-92-5 | Nonadecane | 53.67±13.53^b^ | 78.32±20.40^a^ | ND | ND | 68.31±5.90^ab^ | ND | ND |
|  |  |  |  |  |  |  |  |  |
| 1120-21-4 | Undecane | ND | ND | ND | 316.64±39.82^a^ | ND | ND | ND |
|  |  |  |  |  |  |  |  |  |
|  | **Phenolics** | **0** | **0** | **0** | **0** | **0** | **2 Kinds** | **1 Kind** |
| 1195-09-1 | 2-Methoxy-5-methylphenol | ND | ND | ND | ND | ND | 1374.48±238.10^a^ | ND |
|  |  |  |  |  |  |  |  |  |
| 2785-89-9 | 4-Ethyl-2-methoxyphenol | ND | ND | ND | ND | ND | 476.46±69.86^a^ | 35.73±9.83^b^ |
|  |  |  |  |  |  |  |  |  |
|  | **Acid** | **0** | **0** | **1 Kind** | **1 Kind** | **0** | **1 Kinds** | **0** |
| 64-19-7 | Acetic acid | ND | ND | 531.18±481.88^b^ | 1035.68±188.36^a^ | ND | 763.79±127.81^ab^ | ND |
|  |  |  |  |  |  |  |  |  |
|  | **Others** | **2 Kinds** | **2 Kinds** | **2 Kinds** | **2 Kinds** | **1 Kind** | **2 Kinds** | **2 Kinds** |
| 3777-69-3 | 2-Pentylfuran | 79.83±6.30^e^ | 231.89±24.61^b^ | 360.45±68.48^a^ | 216.82±10.40^bc^ | 211.01±26.98^bc^ | 122.89±28.10^de^ | 154.46±36.37^cd^ |
|  |  |  |  |  |  |  |  |  |
| 24683-00-9 | 2-Methoxy-3-isobutyl pyrazine | 179.32±28.34^b^ | 227.14±57.95^a^ | 158.65±17.24^bc^ | 113.47±7.76^cd^ | ND | 89.95±17.02^d^ | 127.61±72.37^cd^ |
|  |  |  |  |  |  |  |  |  |

Note: Different letters in the same row indicate significant differences (*p* < 0.05, Confidence Interval ：CI_95%_) and “ ND” indicates that the substance was not detected.


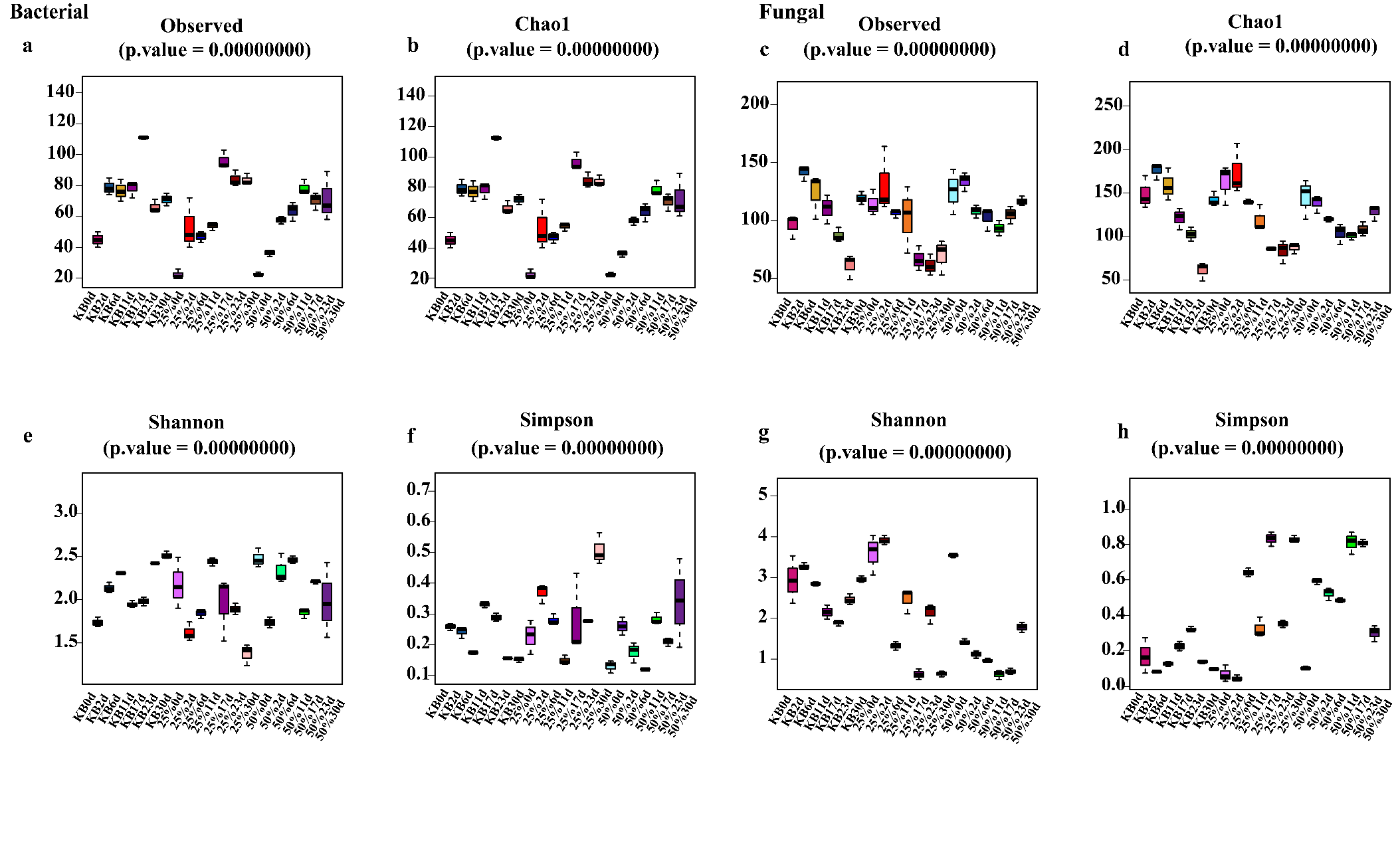


**Figure. S1**. α-diversity during the fermentation of pickled peppers in three groups. (a) Bacterial Observed index; (b) bacterial Chao 1 index; (c) fungal Observed index; (d) fungal Chao 1 index; (e) bacterial Shannon index; (f) bacterial Simpson index; (g) fungal Shannon index; and (h) fungal Simpson index.


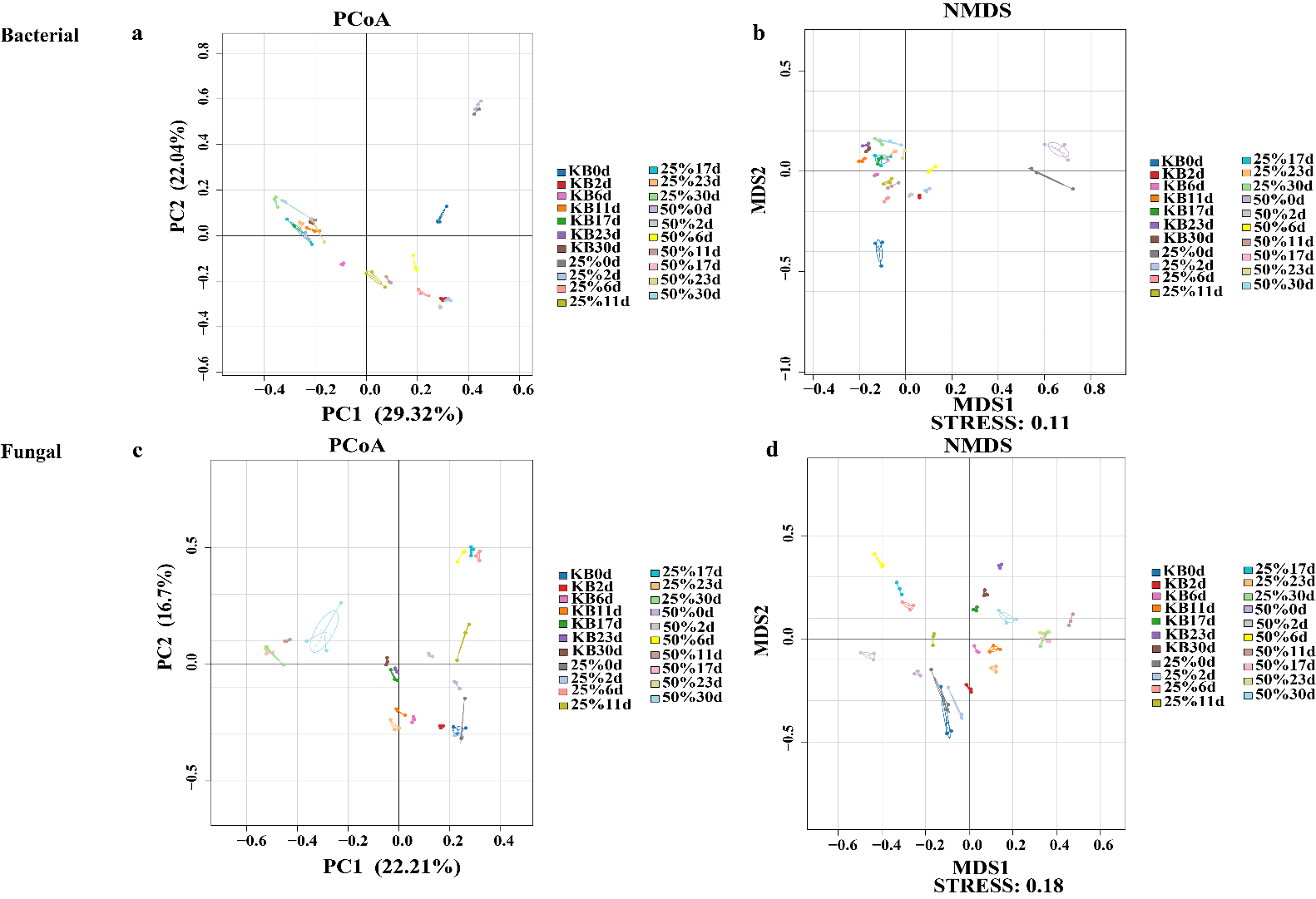


**Figure. S2**. β-diversity during the fermentation of pickled peppers in three groups. (a) Bacterial PCoA plot, (b) bacterial NMDS plot, (c) fungal PCoA plot, (d) fungal NMDS plot.


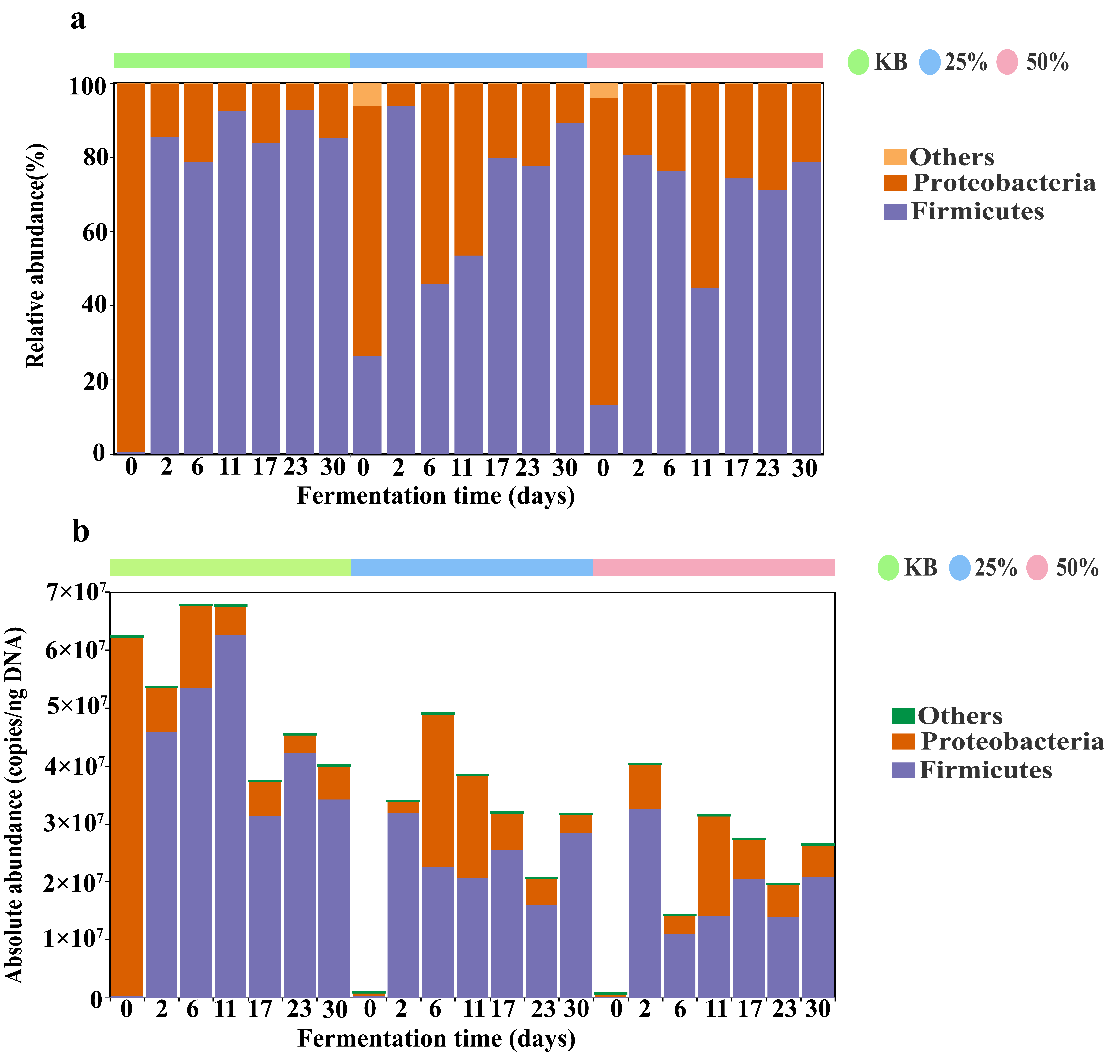


**Fig.S3**: Composition of bacterial communities in three pickled pepper groups. (a) Relative abundance of bacteria at the phylum level; (b) Absolute abundance of bacteria at the phylum level.


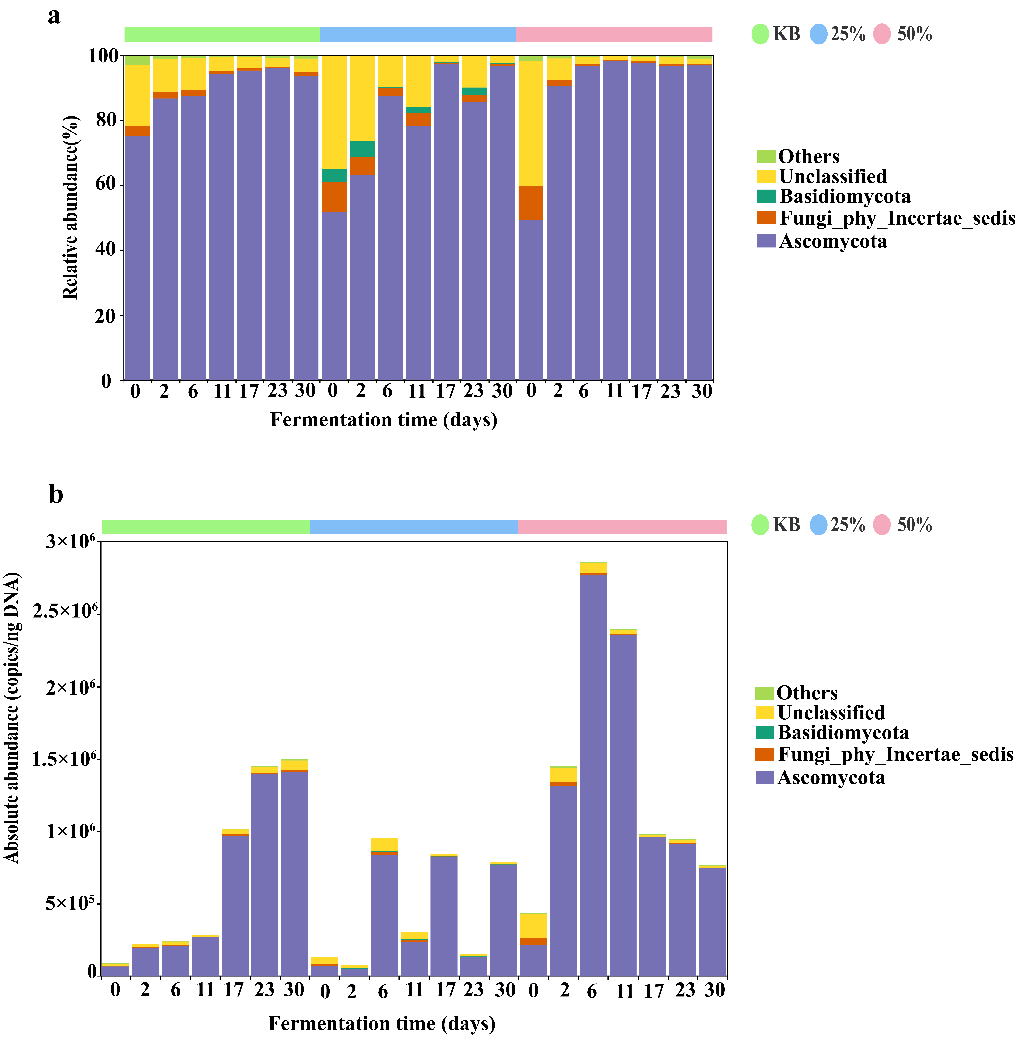


**Fig.S4**: Composition of fungal communities in three pickled pepper groups. (a) Relative abundance of fungi at the phylum level; (b) Absolute abundance of fungi at the phylum level.


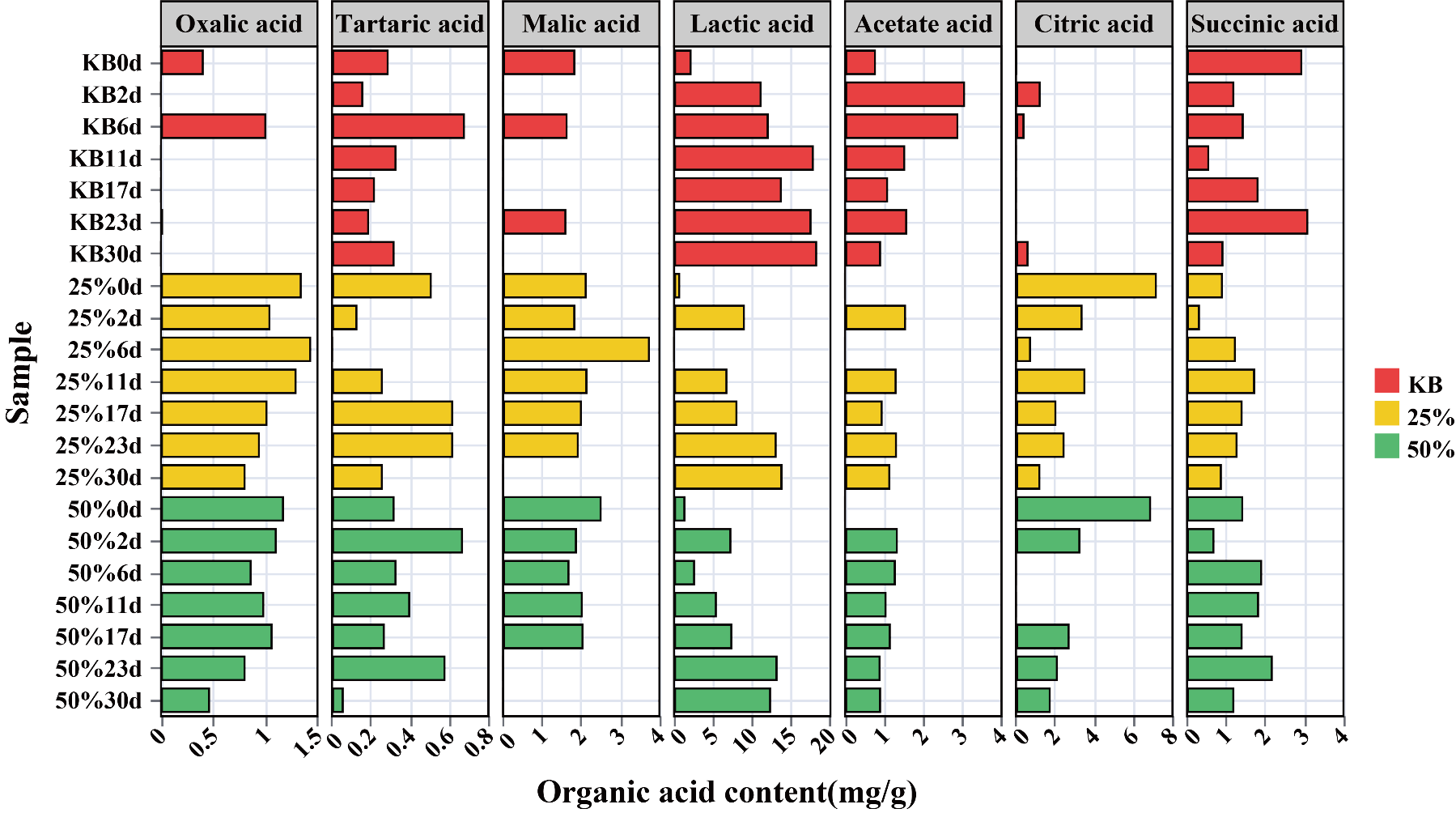


**Fig.S5**: Organic acid content during fermentation of three pickled pepper groups. The KB group is denoted in red, the 25% beer-added group in yellow, and the 50% beer-added group in green.


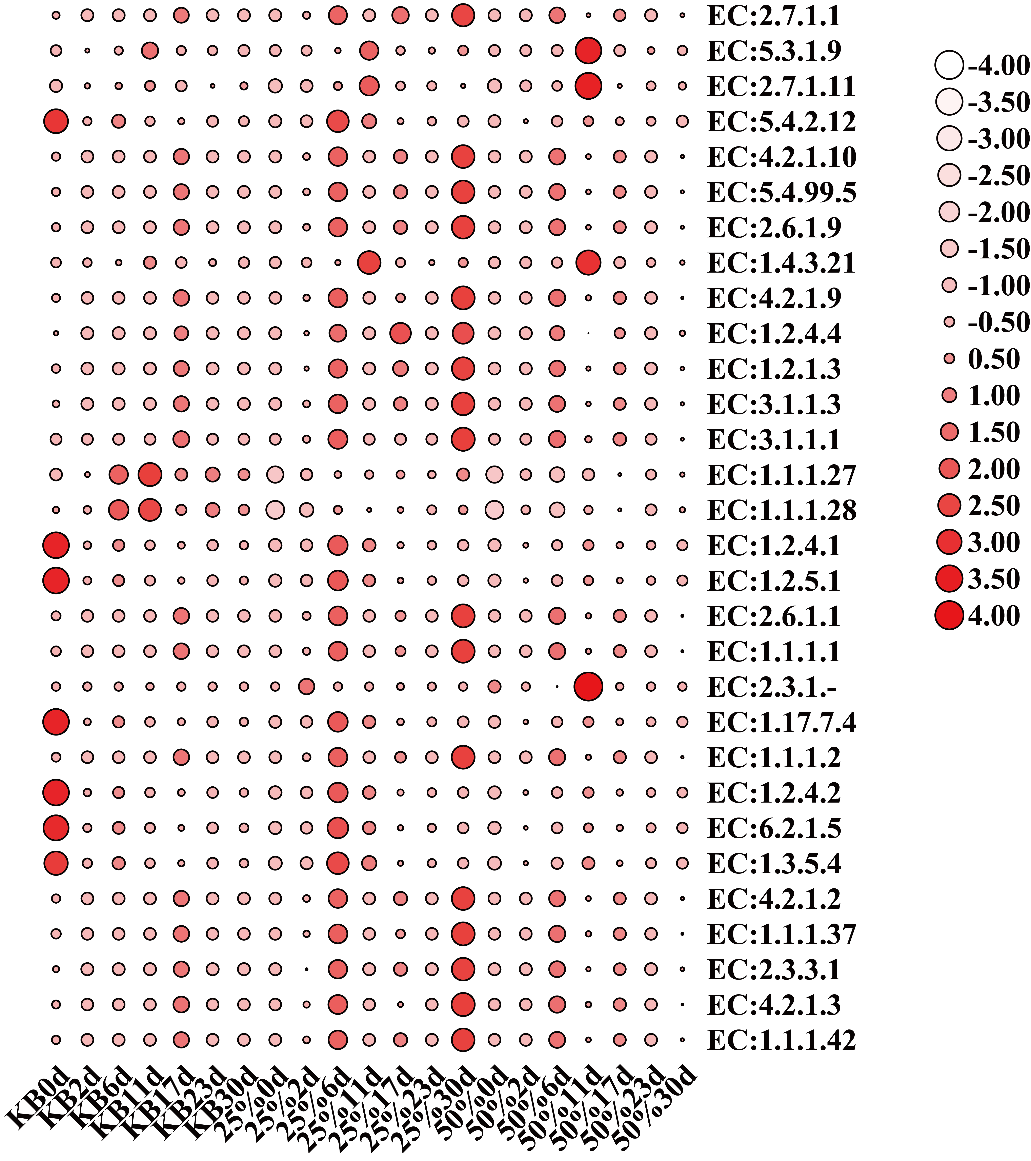


**Fig. S6**. Heatmap of the abundance changes of key functional enzymes predicted by PICRUSt2.
